# Alzheimer’s disease-associated β-amyloid does not protect against herpes simplex virus 1 infection in the mouse brain

**DOI:** 10.1016/j.jbc.2021.100845

**Published:** 2021-05-28

**Authors:** Olga Bocharova, Narayan P. Pandit, Kara Molesworth, Aidan Fisher, Olga Mychko, Natallia Makarava, Ilia V. Baskakov

**Affiliations:** 1 Center for Biomedical Engineering and Technology, University of Maryland School of Medicine, Baltimore, Maryland, USA; 2 Department of Anatomy and Neurobiology, University of Maryland School of Medicine, Baltimore, Maryland, USA

**Keywords:** Alzheimer’s disease, herpes simplex virus 1, Aβ aggregates, 5XFAD mice, microglia, astrocytes, amyloid precursor protein, Aβ, β-amyloid, AD, Alzheimer’s disease, APP, amyloid precursor protein, CNS, central nervous system, GFAP, glial fibrillary acidic protein, HHV6 and HHV7, human herpesviruses 6 and 7, HSE, herpes simplex encephalitis, HSV-1, herpes simplex virus 1, IC, intracranially, LD, lethal dose, MOI, multiplicity of infection, PFU, plaque-forming unit, PS1, presenilin 1, WT, wild-type

## Abstract

Alzheimer’s disease (AD) is a devastating fatal neurodegenerative disease. An alternative to the amyloid cascade hypothesis is that a viral infection is key to the etiology of late-onset AD, with β-amyloid (Aβ) peptides playing a protective role. In the current study, young 5XFAD mice that overexpress mutant human amyloid precursor protein with the Swedish, Florida, and London familial AD mutations were infected with one of two strains of herpes simplex virus 1 (HSV-1), 17syn+ and McKrae, at three different doses. Contrary to previous work, 5XFAD genotype failed to protect mice against HSV-1 infection. The region- and cell-specific tropisms of HSV-1 were not affected by the 5XFAD genotype, indicating that host–pathogen interactions were not altered. Seven- to ten-month-old 5XFAD animals in which extracellular Aβ aggregates were abundant showed slightly better survival rate relative to their wild-type (WT) littermates, although the difference was not statistically significant. In these 5XFAD mice, HSV-1 replication centers were partially excluded from the brain areas with high densities of Aβ aggregates. Aβ aggregates were free of HSV-1 viral particles, and the limited viral invasion to areas with a high density of Aβ aggregates was attributed to phagocytic activity of reactive microglia. In the oldest mice (12–15 months old), the survival rate did not differ between 5XFAD and WT littermates. While the current study questions the antiviral role of Aβ, it neither supports nor refutes the viral etiology hypothesis of late-onset AD.

Alzheimer’s disease (AD) is a devastating fatal neurodegenerative disease, which is estimated to affect 5.8 million Americans in 2020. The vast majority of AD cases are late-onset, which is believed to be sporadic in origin. Over the years, a number of gene variants that increase the susceptibility to late-onset AD have been identified (1), yet our understanding of the etiology of the disease still remains limited. According to the amyloid cascade hypothesis, the pathological cascade of events leading to AD is triggered by aggregation and accumulation of Aβ peptides 1 to 40 and 1 to 42, the proteolytic fragments of amyloid precursor protein (APP) (2, 3).

An alternative to the amyloid cascade hypothesis is the hypothesis that a viral or microbial infection of the central nervous system (CNS) is an essential component of the etiology of late-onset AD (4, 5, 6). According to this hypothesis, which is known as the antimicrobial or antiviral protection hypothesis, Aβ peptides possess antimicrobial and antiviral effects and are produced by the CNS as a defense mechanism (7, 8, 9, 10). Viral and microbial pathogens trigger a pathological cascade, presumably by seeding of Aβ fibrils or aggregates, which can entrap and neutralize CNS pathogens (7, 9, 11). While individuals who experienced a single viral challenge or have a latent infection might not be at high risk for late-onset AD, recurrent reactivation of a latent infection, or an accumulative effect of low-grade chronic infection, and/or multiple viral challenges over the lifetime place an individual at high risk for late-onset AD. Such risk is realized in individuals expressing the high-risk AD variants of *APOE*, *TREM2*, *CR1*, *CD33*, and other genes, many of which are associated with innate immunity. A number of pathogens including *Chlamydia pneumonia* (12, 13, 14, 15), *Borrelia spirochetes* (16, 17), Herpes zoster (18), human herpesviruses 6 and 7 (HHV6 and HHV7) (19), and herpes simplex virus 1 (HSV-1) (4, 20, 21) have been linked to late-onset AD. Among a broad range of pathogens, HSV-1 has emerged as one of the leading pathogens linked to late-onset AD in a number of independent studies (reviewed in (4, 22, 23)).

A recent study by Dudley and coauthors examined hundreds of brains across multiple datasets and reported a greater abundance of HHV6 or HHV7 RNA and DNA in the brains of late-onset AD individuals relative to controls (24). This study suggested that herpes viruses drive the production of Aβ peptides (24). Simultaneously, the work by Eimer and coauthors showed that Aβ peptides protect neurons in 3D cultures and prolong the survival of young 5XFAD mice infected with HSV-1 (7). A recent study reanalyzed the data from Dudley’s work and concluded that the statistical analysis in Dudley’s study was too weak to prove a link between viral load and AD (25). Moreover, the most recent work by Jacobson and coauthors showed no differences between postmortem AD and control human brains with respect to viral RNA/DNA load (26). While Jacobson’s study questioned previous results, a viral role in the etiology of AD was not ruled out by the new findings.

Upon isolation of multiple strains of HSV-1 (strain is defined as a plaque-purified clinical isolate (27)), a genomic diversity of HSV-1 has been demonstrated (28). HSV-1-strain-specific features were shown to dictate important aspects of host–pathogen interaction including the median lethal dose (LD_50_) value, reactivation from latency, and possibly, cell tropism (27, 29, 30, 31). In order to examine whether the protective role of the 5XFAD genotype is dictated by the strain identity of HSV-1, in the current study we employed the approach introduced by Eimer and coauthors (7). This approach involves testing 5XFAD mice that overexpress mutant human APP with the Swedish (K670N, M671L), Florida (I716V), and London (V717I) familial AD mutations along with human presenilin 1 (PS1) harboring two mutations, M146L and L286V (32), to resist acute HSV-1 infection. Contrary to previous results (7), in the current work, the 5XFAD genotype failed to protect the mice upon challenges with two HSV-1 strains, 17syn+ and McKrae. The region-specific or cell-specific tropisms of HSV-1 strains were not affected by the 5XFAD genotype, when compared with wild-type (WT) littermate controls, suggesting that the host–pathogen interactions were not affected by APP overexpression. Young 5XFAD mice that survived acute herpes simplex encephalitis (HSE) cleared HSV-1 infection without triggering extracellular Aβ aggregation in the brain areas vulnerable to HSV-1. Aged, 7- to 10-month-old 5XFAD animals, in which extracellular Aβ aggregates were abundant, showed a delay and slightly better survival rate relative to WT mice along with partial exclusion of HSV-1 replication from brain areas with high densities of Aβ aggregates. In 7- to 10-month-old 5XFAD mice, Aβ aggregates were free of HSV-1 viral particles, whereas reduced activity of HSV-1 in areas with Aβ aggregates was explained by the presence of reactive microglia primed for phagocytosis. In 12- to 15-month-old groups, the survival rate did not differ between 5XFAD and WT littermates. In summary, the current results question an antiviral role for Aβ. Nevertheless, the current work neither supports nor refutes the hypothesis of the viral etiology of late-onset AD.

## Results

### Lack of protective effect of the 5XFAD genotype in young mice

**Figure 1:**
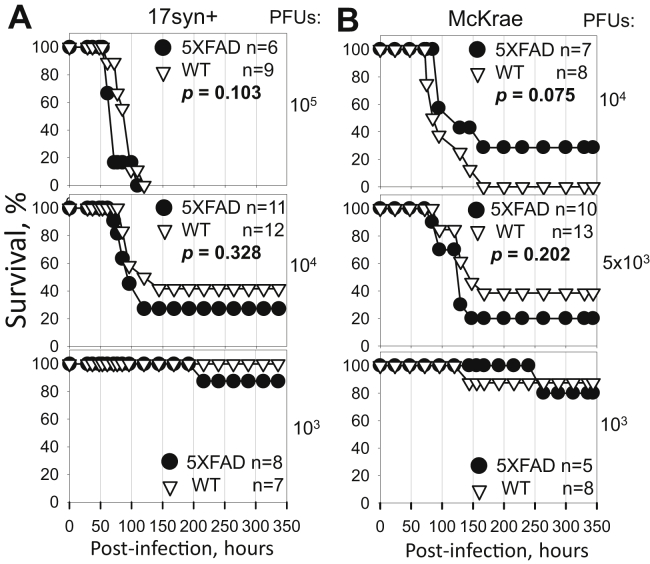
**Dose–response of young 5XFAD mouse model to HSV-1 challenge.** Survival curves for 5- to 6-week-old 5XFAD and wild-type littermate (WT) mice challenged *via* IC injections with 10^5^, 10^4^, or 10^3^ PFUs of 17syn+ strain per mouse (*A*); or 10^4^, 5 × 10^3^, or 10^3^ PFUs of McKrae strain per mouse (*B*). 5XFAD and WT littermate mice were caged together in random ratios. Individual plots show independent experiments with number (*n*) of animals of each genotype indicated. Statistical significance (*p*) was calculated using the log-rank (Mantel–Cox) test.

For preparing HSV-1 inoculation stocks, two commonly used HSV-1 strains, 17syn+ and McKrae, were propagated in Vero cells and titrated using the same cell line. The McKrae strain was shown to be more neurovirulent and had a lower LD50 value relative to 17syn+ (27, 31, 33). To test whether the 5XFAD genotype has protective effects against HSV-1 encephalitis, we used male and female 5XFAD and littermate B6SJL (WT) control mice of the same age as in previous studies (7), *i.e.*, 5- to 6-week-old. Two strains of HSV-1, either 17syn+ or McKrae, were administered intracranially (IC) to examine the strain specificity of the effects. Three doses of each strain were tested: 10^5^, 10^4^, and 10^3^ PFUs (plaque-forming units) for 17syn+ and 10^4^, 5 × 10^3^, and 10^3^ PFUs for McKrae, where the highest and the lowest doses were selected to be above and below of the LD_50_ values for each HSV-1 strain, respectively. As expected, the highest doses resulted in the highest mortality rates (Fig. 1, *A* and *B*, top). Reducing the viral dose increased the survival rates and prolonged the incubation times of nonsurvivors in both 5XFAD and WT cohorts challenged with both HSV-1 strains (Fig. 1, *A* and *B*). Out of six experimental conditions tested, 5XFAD mice showed higher survival rates relative to the WT littermates only in one condition: animals challenged with 10^4^ PFUs of McKrae (Fig. 1*B*, upper panel); however, the difference between 5XFAD and WT cohorts lacked statistical significance. Moreover, lowering the inoculation dose twofold, from 10^4^ PFUs to 5 × 10^3^ PFUs (Fig. 1*B*, middle panel), reversed the survival yield between 5XFAD and WT littermates suggesting that minor variations in survival yield might be due to stochastic variations between experiments.

Because a previous study that documented protective effects of the 5XFAD genotype employed only female mice (7), and because female 5XFAD mice express higher levels of APP and generate higher levels of Aβ relative to male 5XFAD mice (32, 34), the survival curves were reanalyzed for females only. Survival of females showed the same pattern as survival of males and females combined (Fig. S1, *A* and *B*) and, again, showed no statistically significant differences between 5XFAD and WT cohorts. In summary, these experiments employing two HSV-1 strains, 17syn+ and McKrae, revealed a lack of protective effect of the 5XFAD genotype against HSV-1 encephalitis.

### Region-specific tropism of HSV-1 is not altered in 5XFAD mice

**Figure 2:**
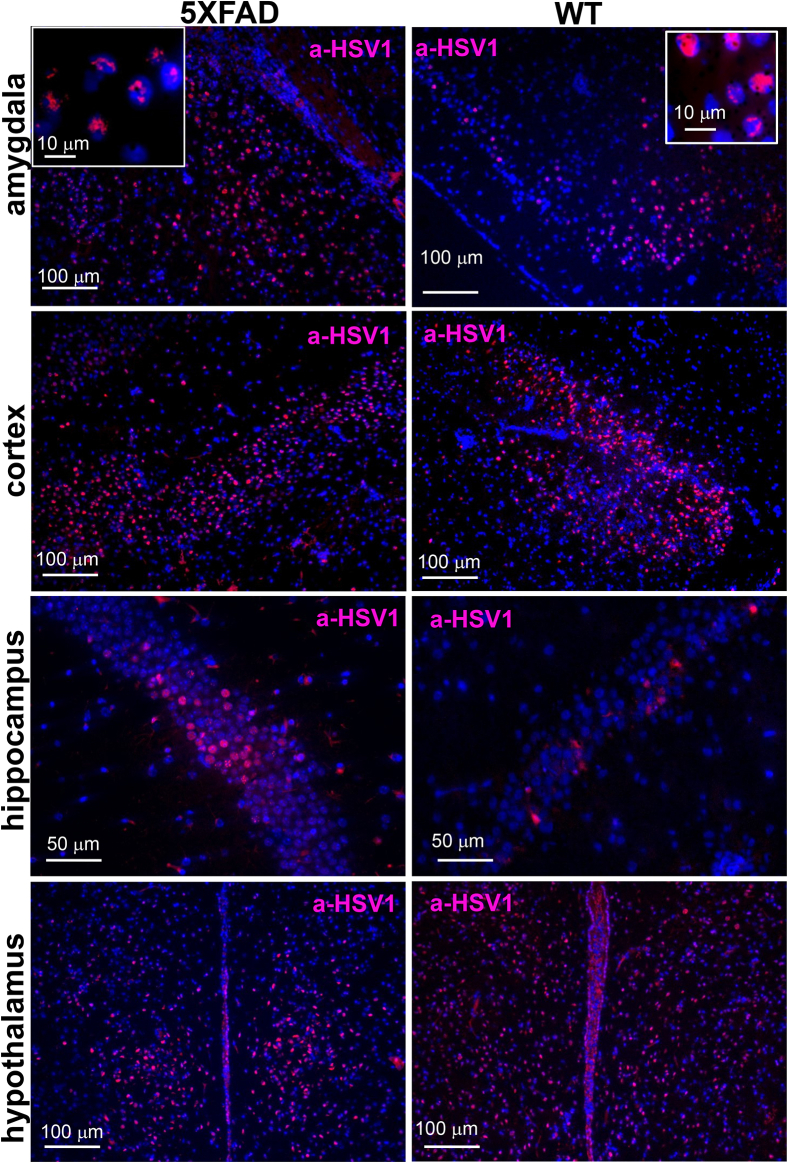
**Region-specific tropism of HSV-1 is not altered in young 5XFAD mice.** Immunostaining for HSV-1 replication centers (a-HSV1 antibody, *red*) and cell nuclei (DAPI, *blue*) in 6-week-old 5XFAD (*n* = 6 mice) and WT littermates (*n* = 7 mice) that did not survive IC challenges with 10^5^ PFUs of 17syn+.

To test whether high expression levels of the disease-associated APP variant in 5XFAD mice alter HSV-1 tropism, brains of 5XFAD mice and WT littermates were analyzed using immunostaining for HSV-1. Infected cells were detected using anti-HSV1 (referred to as a-HSV1) antibodies that stain HSV-1 replication centers (35). Depending on replication stage, a-HSV1 staining displays granular or diffuse staining patterns within nuclei of infected cells (Fig. 2) (35). Hippocampus, hypothalamus, cortex, and amygdala were consistently found to be among the most severely infected brain regions in both 5XFAD and WT control animals that succumbed to acute diseases (Fig. 2 and Fig. S2). No a-HSV1 staining was found in age-matched noninfected 5XFAD mice (Fig. S2). While variations in regional distribution of HSV-1replication sites were found within animals that succumbed to viral infection, the most affected brain regions remained the same in animals of both genotypes.

All animals that survived challenges with 10^4^ and 10^3^ PFUs of 17syn+ or 10^4^, 5 × 10^3^, and 10^3^ PFUs of McKrae were examined at the endpoint of the experiment (336 h postinoculation, Fig. 1, *A* and *B*) for the presence of HSV-1 using staining with a-HSV1. However, regardless of the genotype, no signs of viral replication were found, suggesting that animals that survived the acute stages of simplex HSE completely cleared the viral infection (data not shown).

### Cell-specific tropism of HSV-1 is not altered in 5XFAD mice

**Figure 3:**
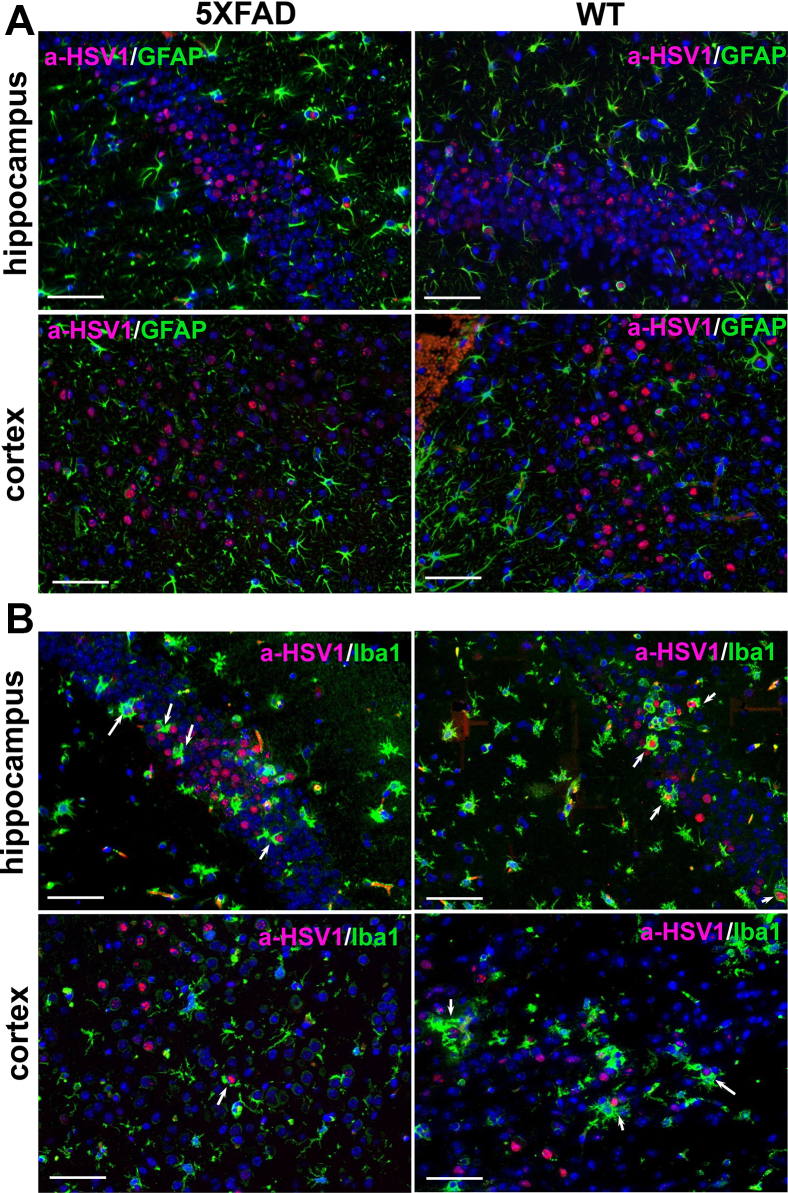
**Cell-specific tropism of HSV-1 is not altered in young 5XFAD mice.***A*, coimmunostaining for HSV-1 replication centers (a-HSV1 antibody, *red*), astrocytes (anti-GFAP antibody, *green*, Cell Signaling Technology), and cell nuclei (DAPI, *blue*) in 6-week-old 5XFAD (*n* = 5 mice) and WT littermates (*n* = 5 mice) that did not survive IC challenges with 10^4^ PFUs of 17syn+. *B*, coimmunostaining for HSV-1 replication centers (a-HSV1 antibody, *red*), microglia (anti-Iba1 antibody, *green*), and nuclei (DAPI, *blue*) in 5XFAD (*n* = 5 mice) and WT littermates (*n* = 5 mice) that did not survive IC challenges with 10^4^ PFUs of 17syn+. *Arrows* point at microglia engulfing HSV-1-infected cells. Scale bars = 50 μm.

*In vitro*, HSV-1 infects different cell types including astrocytes, microglia, and oligodendrocytes, whereas *in vivo* the virus predominantly invades neurons (36, 37, 38, 39, 40). To test whether high expression of the human APP variant in neurons protects neuronal cells from invasion and alters cell tropism, brain slices were coimmunostained for HSV-1 replication sites and for glial fibrillary acidic protein (GFAP), a marker for astrocytes, or for Iba1, a microglia-specific marker. In both genotypes, 5XFAD and WT littermate controls, the vast majority of HSV-1-infected cells were neurons. Extremely rare HSV-1-infected astrocytes and no HSV-1-positive microglia were found (Fig. 3, *A* and *B*). In both the 5XFAD and WT cohorts, reactive microglia were often found in close vicinity to HSV-1-infected neurons or were engulfing infected cells (Fig. 3*B*). To summarize, no differences with respect to the infected cell types or infected brain areas were observed between 5XFAD and WT littermates.

### HSV-1 infects both APP-positive and -negative neurons

**Figure 4:**
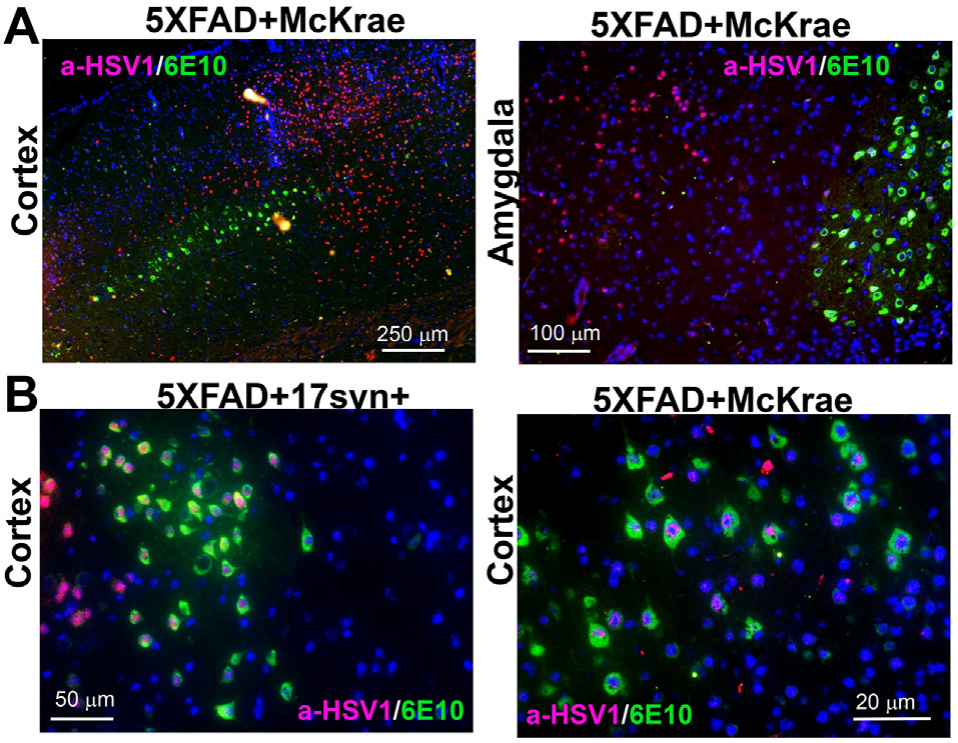
**HSV-1 Infects both APP-positive and -negative neurons.***A*, coimmunostaining for HSV-1 replication centers (a-HSV1 antibody, *red*), APP (6E10 antibody, *green*), and nuclei (DAPI, *blue*) in 6-week-old 5XFAD (*n* = 6 mice) challenged IC with 5 × 10^3^ PFUs of McKrae. *B*, coimmunostaining for HSV-1 replication centers (a-HSV1 antibody, *red*), APP (6E10 antibody, *green*), and nuclei (DAPI, *blue*) in 5XFAD mice challenged IC with 10^5^ PFUs of 17syn+ (*left panel*, *n* = 2 mice) or 10^4^ PFUs of McKrae (*right panel*, *n* = 5 mice).

Next, we examined whether cells expressing high levels of APP are protected from HSV-1 invasion. In 5XFAD mice, APP is expressed under the Thy1 promoter and only a subpopulation of cells with active Thy1 promoters express high levels of APP. Consistent with previous studies (32), in the current work, APP was found primarily in pyramidal neurons in cortical layer 5, subiculum, as well as pyramidal neurons and granule cells of the hippocampus and amygdala nuclei. Under low magnification, the sites of active HSV-1 replication seemed to be excluded from the areas expressing high levels of APP (Fig. 4*A*). It is not clear whether this effect was due to actual antiviral effects of APP or intrinsic tropism of HSV-1 to a subpopulation of neurons with low Thy1 activity. High-magnification imaging revealed HSV-1 replication sites in both APP-positive and -negative neurons (Fig. 4*B*). In 5XFAD mice, elevated levels of intraneuronal Aβ42 deposition were observed starting after 1.5 months of age (32). Because 6E10 antibody used in this experiment stains both APP and Aβ peptides, the presence of Aβ peptides in APP-positive cells cannot be excluded. Nevertheless, because in young 5XFAD animals, intracellular 6E10 signal is attributed primarily to APP, the current result demonstrates that HSV-1 can infect both APP-positive and -negative neurons.

### HSV-1 infection does not induce Aβ aggregates in young 5XFAD mice

In previous studies, positive coimmunostaining for HSV-1 and Aβ plaques was detected in 5XFAD survivors, 3 weeks postinoculation (7). For testing whether HSV-1 triggered formation of extracellular Aβ aggregates in the current study, 5XFAD mice that survived IC challenges with 17syn+ or McKrae delivered to 5- to 6-week-old mice were examined by coimmunostaining for Aβ using 6E10 antibody and HSV-1 replication centers using a-HSV1. Along with APP and Aβ peptides, 6E10 antibody stains extracellular Aβ aggregates including plaques. Previous studies established that in the 5XFAD model, the first extracellular aggregates appears in 2-month-old mice in specific brain areas—subiculum, somatomotor, and somatosensory parts of the cortex (32). To test whether HSV-1 induces Aβ aggregate formation, we focused on two specific brain areas—hippocampus and primary motor cortex, which were aggregate-free in uninfected age-matched 5XFAD mice but showed extensive replication of the virus during the acute stages of the infection. No extracellular Aβ aggregates were detected in the hippocampus or primary motor cortex in 5XFAD survivors 2 or 7.5 weeks postinoculation (Fig. S3, *A* and *B*). Noninoculated, aged 5XFAD mice, used as positive controls of Aβ staining, showed extracellular Aβ aggregates (Fig. S3*C*). As mentioned above, in 5XFAD mice that survived HSV-1 infection, no HSV-1 replication centers were found including within those brain areas (primary motor cortex and hippocampus), where intensive HSV-1 replication was seen during acute stages of the infection (Fig. S3, *A* and *B*). These results suggest that HSV-1 was cleared in 5XFAD survivors without triggering Aβ deposition.

### Lack of protective effect of Aβ in aged 5XFAD mice

**Figure 5:**
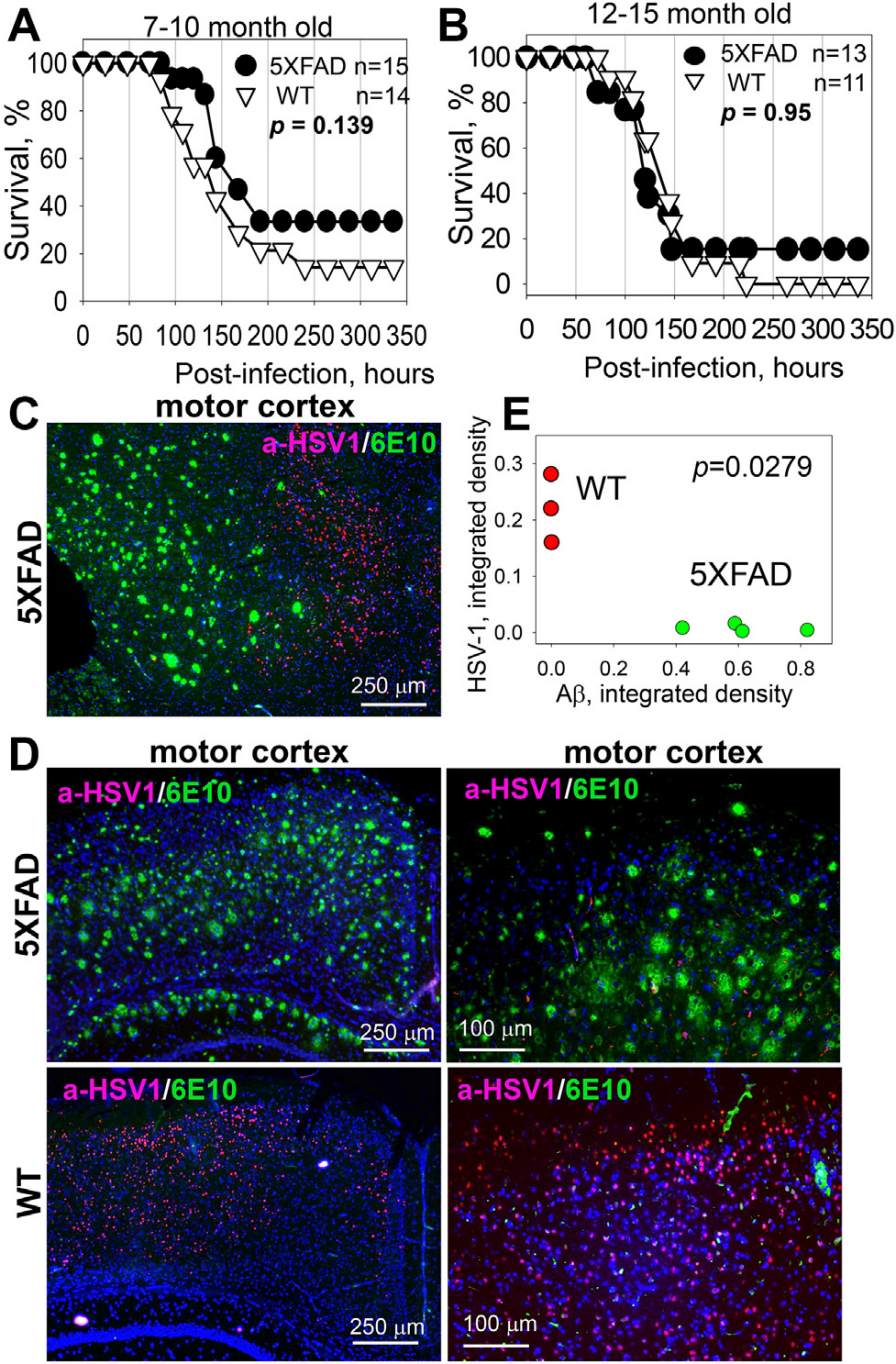
**Lack of protective effect of Aβ in aged 5XFAD mice.***A* and *B*, survival curves for 7- to 10-month-old (*A*) or 12- to 15-month-old (*B*) 5XFAD and WT littermate mice challenged IC with 5 × 10^3^ PFUs of McKrae per mouse. 5XFAD and WT mice were caged together in random ratios. Statistical significance (*p*) was calculated using the log-rank (Mantel–Cox) test. *C* and *D*, coimmunostaining for HSV-1 replication centers (a-HSV1 antibody, *red*), Aβ aggregates (6E10 antibody, *green*), and nuclei (DAPI, *blue*) in 7- to 10-month-old 5XFAD (*n* = 9 mice) and WT littermates (*n* = 4 mice), which were challenged IC with 5 × 10^3^ PFUs of McKrae and succumbed to acute herpes simplex encephalitis within 96 to 200 h postinoculation. *E*, analysis of integrated density of HSV-1 replication centers as a function of integrated density of Aβ aggregates in the motor cortices of McKrae-inoculated 5XFAD (*n* = 4 mice, *green*) and WT littermates (*n* = 3 mice, *red*). Integrated density of HSV-1 immunostaining signal was found to be different in the motor cortices of WT and 5XFAD mice (∗*p*_*adj*_ = 0.0279, calculated using an ordinary two-way ANOVA with Šidák’s multiple comparisons test).

Previous studies suggested that the antiviral effect of Aβ involves binding and entrapping of HSV-1 by Aβ peptides (7). We reasoned that Aβ aggregates could be more effective than nonaggregated Aβ in trapping viruses. In 5XFAD mice, the first Aβ aggregates are found as early as 2 months of age (32). To test whether existing Aβ aggregates are a prerequisite for the antiviral effect, 7- to 10-month-old 5XFAD mice and WT littermates were challenged IC with 5 × 10^3^ PFUs of the McKrae strain. Combined male and female 5XFAD mice showed a slightly better overall survival rate and, particularly, a better survival rate within the first 140 h postinfection relative to WT littermates (Fig. 5*A*). However, the difference between the 5XFAD and WT cohorts lacked statistical significance. Survival curves for females showed the same trend as survival of combined males and females, yet also lacked statistically significant difference between 5XFAD and WT cohorts (Fig. S4*A*). Analysis of HSV-1 genome copy number in the brains of mice at 120 and 144 h postinoculation revealed a large variation between animals within a group, as well as a lack of statistically significant difference between WT and 5XFAD mice in copy number (Fig. S4*B*).

If preformed Aβ aggregates have any protective effects against HSV-1, we expected that the effect should have been more substantial with older age due to increase in Aβ deposition with age. For testing whether this is the case, an additional experiment was conducted using 12- to 15-month-old 5XFAD and WT littermates, which were challenged IC with 5 × 10^3^ PFUs of McKrae (Fig. 5*B*). However, an increase in the age of animals did not improve the survival rates of 5XFAD relative to WT littermates (Fig. 5*B*). In fact, the survival curves for two genotypes were superimposable. To summarize, these experiments show that in aged groups, preexisting Aβ aggregates do not provide protection from acute HSV-1 infection.

### In aged 5XFAD mice, HSV-1 does not replicate in brain regions with Aβ aggregates

Upon IC challenge with 5 × 10^3^ PFUs of McKrae, a cohort of 7- to 10-month-old 5XFAD mice showed slightly better survival rate in comparison to the WT littermate cohort (Fig. 5*A*). Modest differences in the survival curve seen for these two genotypes suggest differences in response to the HSV-1 infection. Indeed, analysis of HSV-1 replication sites revealed that in 5XFAD mice, HSV-1 infection was suppressed in areas with abundant deposition of Aβ aggregates (Fig. 5, *C* and *D*). The most severe effect was observed in parts of motor cortices, which displayed high densities of Aβ aggregates. While HSV-1 replication sites were abundant in the motor cortices of WT littermates, they were barely detectable in 5XFAD mice (Fig. 5*D*). Quantitative imaging that integrates the signal intensity of HSV-1 replication sites confirmed a statistically significant difference between the densities of HSV-1 replication sites in the motor cortices of 5XFAD and WT littermates (Fig. 5*E*). While extracellular Aβ aggregates affected distribution of HSV-1 replication centers across brain areas, this phenomenon seems to provide only limited protection to 5XFAD mice, as the difference in survival rates between 5XFAD and WT was not significant.

### Lack of association between HSV-1 and Aβ aggregates in aged 5XFAD mice

To test whether the lack of viral replication in areas with a high density of Aβ aggregates was due to entrapping of HSV-1 by Aβ aggregates, we examined colocalization of Aβ with HSV-1 using coimmunostaining with anti-Aβ antibody H31L21 (green channel) and antibody to glycoprotein B of HSV-1 viral envelope [anti-gB clone T111 (red channe)] that was used in previous studies to detect association between HSV-1 and Aβ (7). H31L21 was used for detecting Aβ aggregates, because double staining with 6E10 and gB was not possible.

**Figure 6:**
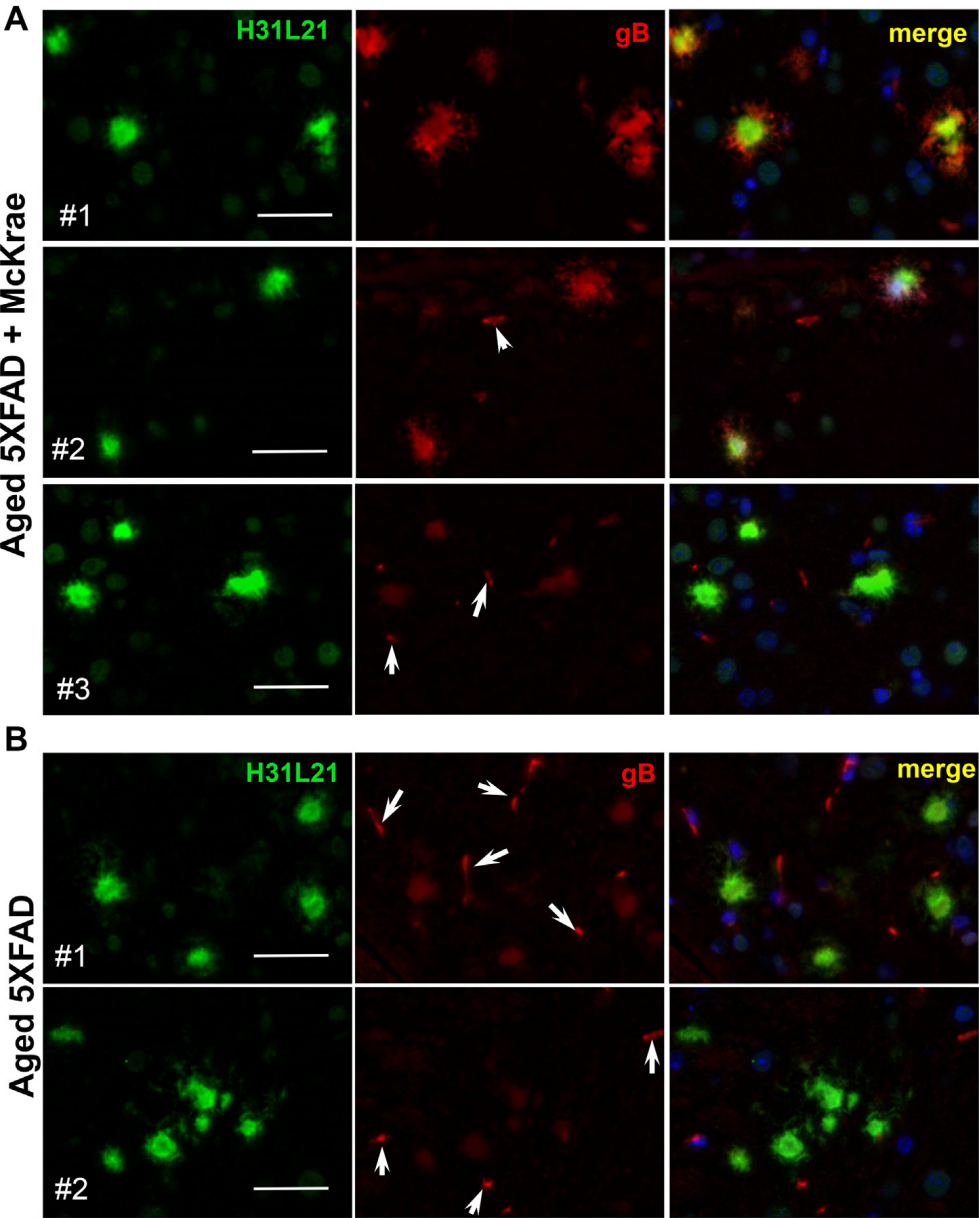
**Coimmunostaining for HSV-1 and Aβ aggregates using anti-gB in aged 5XFAD mice.** Coimmunostaining for Aβ aggregates (H31L21 antibody, *green*), HSV-1 virus (anti-gB antibody, *red*), and nuclei (DAPI, *blue*) in 7- to 10-month-old 5XFAD mice, which were challenged IC with 5 × 10^3^ PFUs of McKrae and succumbed to acute herpes simplex encephalitis within 96 to 200 h postinoculation (*n* = 8 mice) (*A*) and uninoculated age-matched 5XFAD (*n* = 5 mice) (*B*). Animals #1 and #2 in McKrae-challenged group (*A*) showed strong fluorescence signal intensity and elaborate aggregates morphology in the anti-gB channel, whereas animals #3 had the same signal intensity in the anti-gB channel as control group. *Arrows* point at staining of blood vessels. Scale bars = 20 μm.

In 7- to 10-month-old 5XFAD mice inoculated with McKrae, two out of eight 5XFAD mice exhibited a strong signal in the anti-gB channel (Fig. 6*A*, animals #1 and #2), whereas six 5XFAD mice showed weak staining of extracellular Aβ aggregates by anti-gB (Fig. 6*A*, animal #3). Aged noninfected 5XFAD mice serving as negative controls showed weak staining of extracellular Aβ aggregates by anti-gB, which was similar to the intensity observed in six infected 5XFAD animals even after optimization of the staining protocol (Fig. 6*B*). This result suggests that the clone T111 cross-reacts with Aβ aggregates.

**Figure 7:**
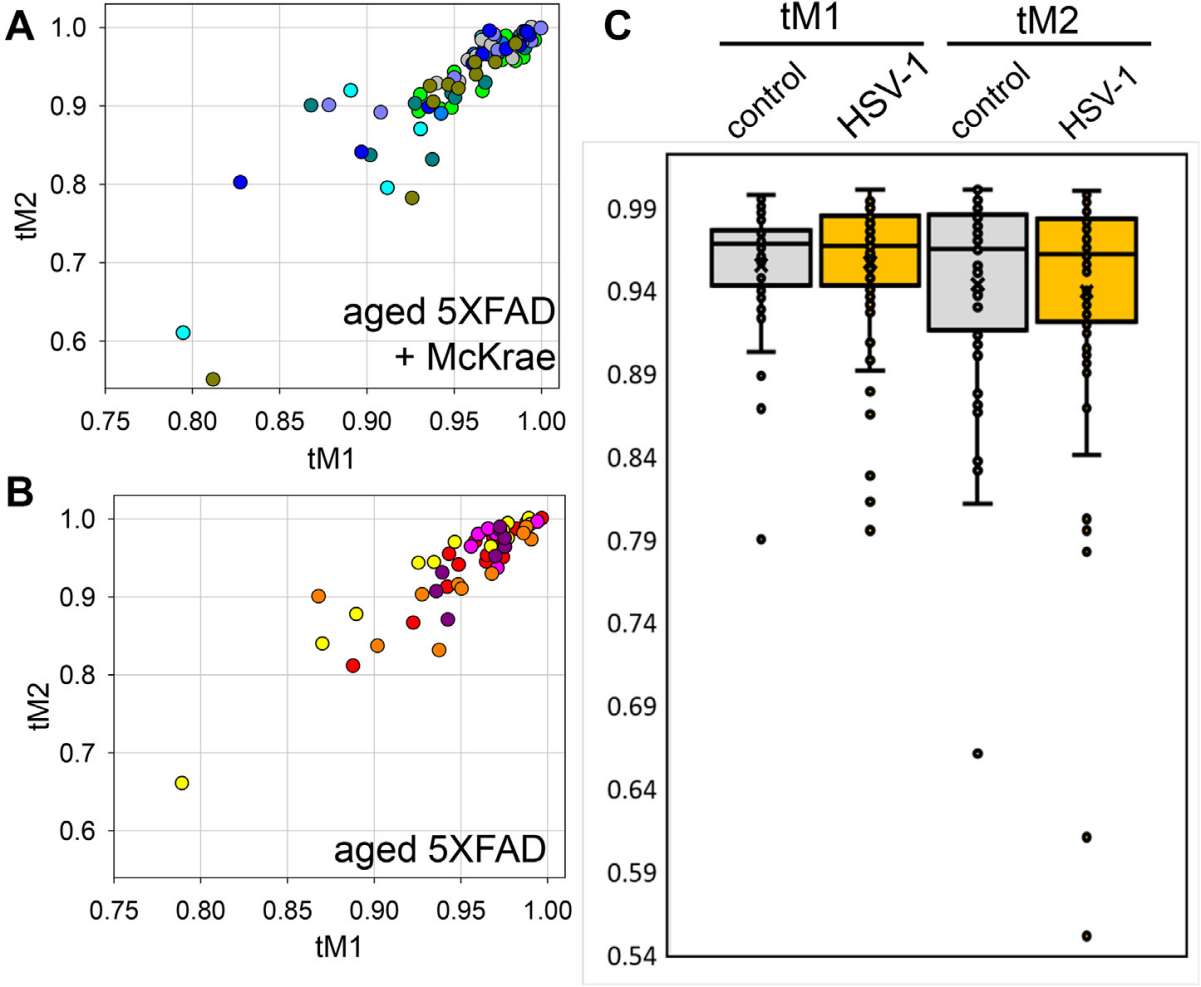
**Analysis of Manders’ overlap coefficients in aged 5XFAD mice.***A* and *B*, distribution of tM1 (*green channel*, Aβ) and tM2 (*red channel*, gB) colocalization coefficients in 5XFAD mice challenged IC with 5 × 10^3^ PFUs McKrae (*n* = 8 mice, 81 individual plaques) (*A*) and age-matched 5XFAD mice (*n* = 5 mice, 55 individual plaques) (*B*). Individual animals are color-coded; each circle represents an individual Aβ aggregate. *C*, significance testing of colocalization data. The box-and-whisker plot of tM1 and tM2 colocalization coefficients in experimental and controls 5XFAD groups are shown in panels *A* and *B*, respectively. The midline of the box-and-whisker plot denotes the median, x represents the mean, and the ends of the box denote the 25th and 75th percentiles.

To answer the question as to whether the signal in infected 5XFAD mice can be attributed entirely to the cross-reactivity of clone T111 or is, at least in part, due to trapping of HSV-1 by Aβ aggregates, we analyzed the degree of colocalization between anti-Aβ and anti-gB antibodies. We reasoned that, if HSV-1 is absent, the cross-reactivity of anti-gB to extracellular Aβ aggregates should produce a similar degree of colocalization between the two channels in both infected and noninfected brains. If the virus is present in Aβ aggregates, the colocalization coefficient between the anti-Aβ and anti-gB channels is expected to be lower in infected *versus* noninfected mice. For quantitative analysis of colocalization, Manders’ Overlap Coefficients tM1 (for anti-Aβ channel) and tM2 (for anti-gB channels) were calculated for individual aggregates from control and experimental 5XFAD groups (Fig. 7, *A* and *B*) (41). Cross-comparison showed lack of differences and very similar distributions of tM1 or tM2 values for uninfected controls and HSV-1 infected groups (Fig. 7*C*). The colocalization analysis supports the conclusion that the signal in the infected 5XFAD group was due to cross-reactivity of clone T111 to Aβ aggregates.

**Figure 8:**
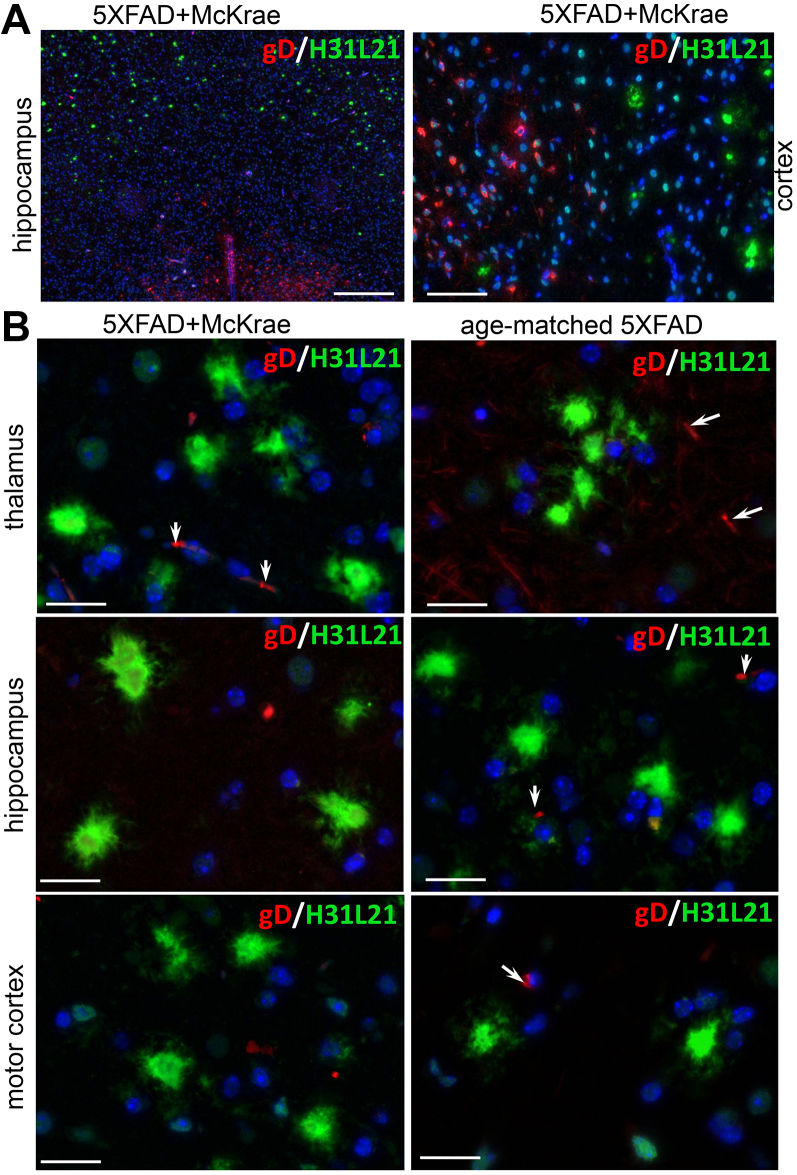
**Coimmunostaining for Aβ aggregates and HSV-1 using anti-gD in aged 5XFAD mice.** Coimmunostaining for Aβ aggregates (H31L21 antibody, *green*), HSV-1 virus (anti-gD antibody, *red*), and nuclei (DAPI, *blue*) in infected 7- to 10-month-old 5XFAD mice (*n* = 8 mice) examined under low (*A*) or high magnifications (*B*), and uninoculated age-matched 5XFAD (*n* = 3 mice) (*B*). 5XFAD mice were challenged IC with 5 × 10^3^ PFUs of McKrae and succumbed to acute herpes simplex encephalitis within 96 to 200 h postinoculation. *Arrows* point at staining of blood vessels. Scale bars = 100 μm in *A* and 20 μm in *B*.

**Figure 9:**
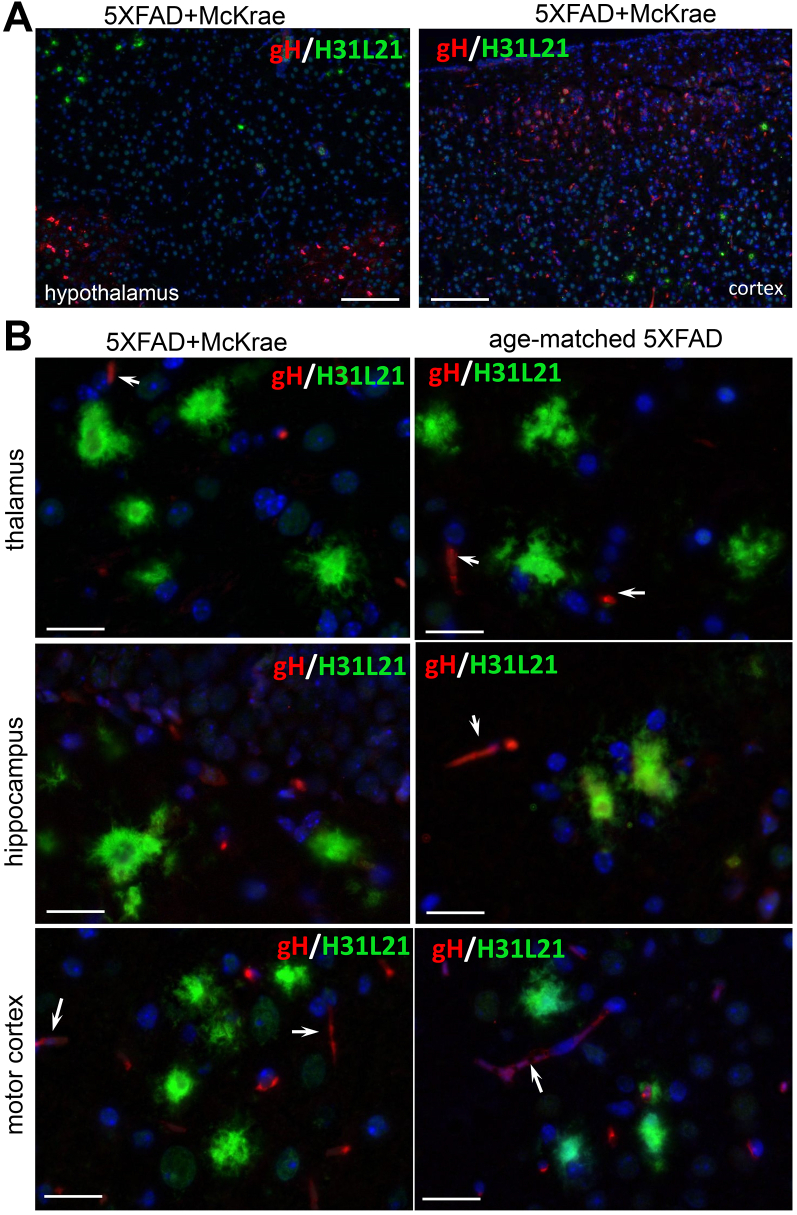
**Coimmunostaining for Aβ aggregates and HSV-1 using anti-gH in aged 5XFAD mice.** Coimmunostaining for Aβ aggregates (H31L21 antibody, *green*), HSV-1 virus (anti-gH antibody, *red*), and nuclei (DAPI, *blue*) in infected 7- to 10-month-old 5XFAD mice (*n* = 8 mice) examined under low (*A*) or high magnifications (*B*), and uninfected age-matched 5XFAD (*n* = 3 mice) (*B*). 5XFAD mice were challenged IC with 5 × 10^3^ PFUs of McKrae and succumbed to acute herpes simplex encephalitis within 96 to 200 h postinoculation. *Arrows* point at staining of blood vessels. Scale bars = 100 μm in *A* and 20 μm in *B*.

To further examine whether HSV-1 binds to Aβ aggregates, we used anti-gD and anti-gH antibodies, which detect glycoproteins D and H of the HSV-1 envelope, respectively, and do not show cross-reactivity to Aβ aggregates. First, coimmunostaining of 5XFAD brains infected with McKrae using a-HSV1 antibody and anti-gD or anti-gH antibodies confirmed that both anti-gD and anti-gH stained the same brain areas and the same cells that were also positive for HSV-1 replication centers (Fig. S5). As expected, a-HSV1 detected replication centers and stained nuclei of infected cells, whereas anti-gD or anti-gH labeled the cytoplasm of a-HSV1-positive cells and pericellular spaces in the vicinity of the a-HSV1-positive cells (Fig. S5). This experiment confirmed that both anti-gD and anti-gH can effectively detect the virus. Next, we determined that immunostaining with anti-gD or anti-gH of aged 5XFAD mice inoculated with McKrae reveals the presence of HSV-1 in mouse brains (Figs. 8*A* and 9A). As found previously, multiple brain areas including cortex, hippocampus, thalamus, and hypothalamus were affected. However, no signs of colocalization between HSV-1 and extracellular Aβ aggregates could be found using anti-gD or anti-gH in any of the brain areas (Fig. 8, *A* and *B* and 9, *A* and *B*). Careful examination of individual Aβ aggregates under high magnification also showed a lack of HSV-1 signal in Aβ aggregates (Figs. 8*B* and 9B). Imaging of noninfected aged 5XFAD mice confirmed the lack of cross-reactivity of anti-gD or anti-gH to Aβ aggregates (Figs. 8*B* and 9*B*). These results argue against the colocalization of HSV-1 with Aβ aggregates.

**Figure 10:**
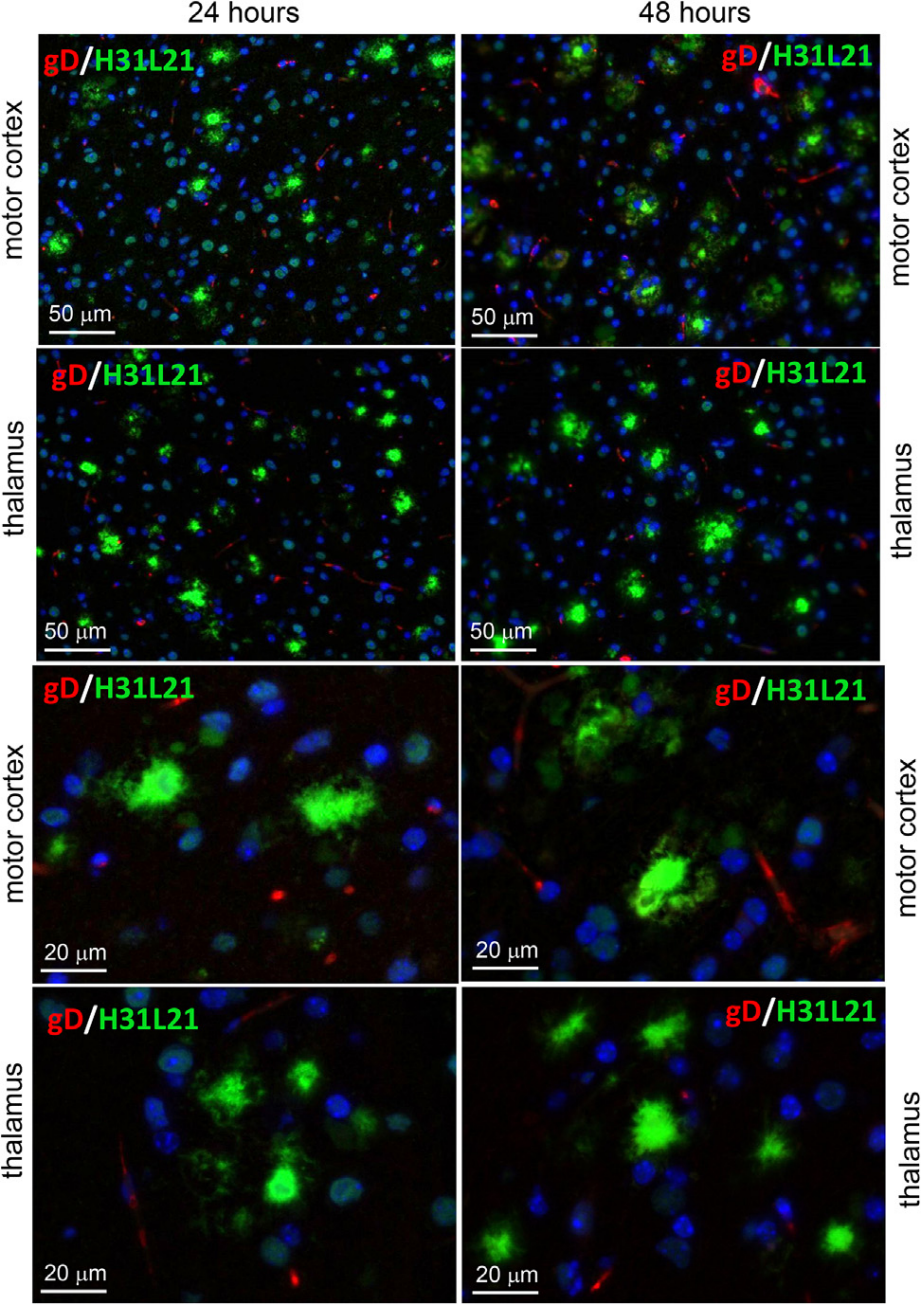
**Coimmunostaining for Aβ aggregates and HSV-1 using anti-gD in aged 5XFAD mice.** Coimmunostaining for Aβ aggregates (H31L21 antibody, *green*), HSV-1 virus (anti-gD antibody, *red*), and nuclei (DAPI, *blue*) in 10-month-old 5XFAD mice challenged IC with 10^4^ PFUs of McKrae examined at 24 h (*n* = 5 mice) or 48 h (*n* = 5 mice) postinoculation. *Arrows* point at staining of blood vessels.

To explain the absence of HSV-1 in extracellular Aβ aggregates, we considered the possibility that the virus could have been entrapped by Aβ aggregates temporarily, but then quickly cleared by microglia. To test whether HSV-1 was present in Aβ aggregates only at the early time points of infection, aged 5XFAD were inoculated with the highest titer of McKrae 10^4^ PFUs used in the current work and examined at 24 or 48 h postinoculation. Coimmunostaining using anti-Aβ antibody H31L21 and anti-gD antibody did not show any signs of colocalization of HSV-1 with Aβ aggregates (Fig. 10). We conclude that extracellular Aβ aggregates do not entrap HSV-1 virus.

### Reactive microglia limits spread of HSV-1 in brain areas with high density of Aβ aggregates

**Figure 11:**
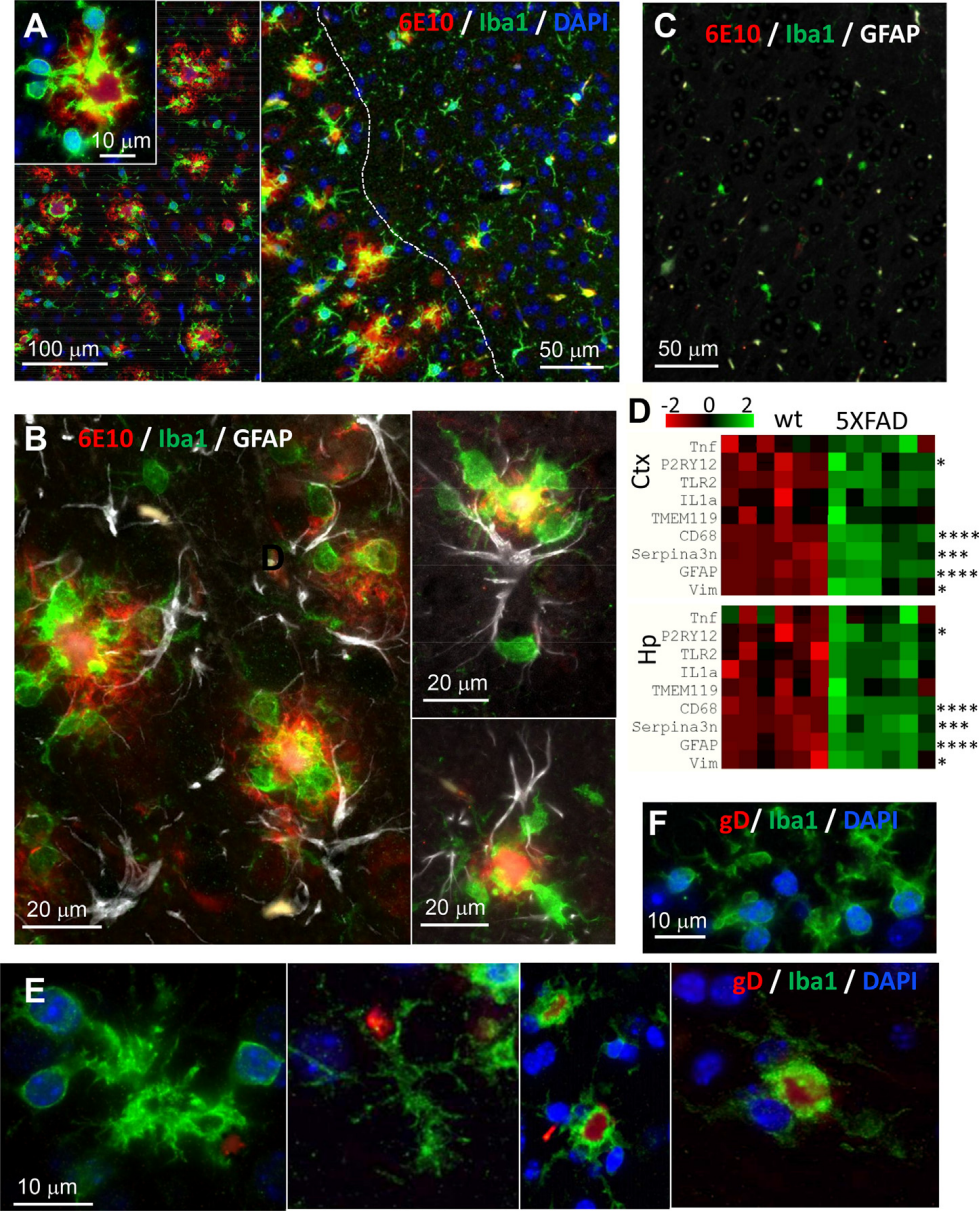
**Reactive microglia are primed for phagocytosis in the areas with high density of Aβ aggregates in aged 5XFAD mice.***A*–*C*, coimmunostaining for Aβ aggregates (6E10 antibody, *red*), reactive microglia (anti-Iba1 antibody, *green*), and nuclei (DAPI, *blue*) (*A*), or Aβ aggregates (6E10 antibody, *red*), reactive microglia (anti-Iba1 antibody, *green*), and reactive astrocytes (anti-GFAP, Millipore, *white*) (*B* and *C*) of motor cortices of 10-month-old 5XFAD mice (*A* and *B*) or WT littermates (*C*). The *dashed line* in panel *A* separates areas with a high density of Aβ aggregates. *D*, heatmap analysis of the expression of genes that reports on reactive state of microglia and astrocytes in cortex (Ctx) and hippocampus (Hp) of 10-month-old 5XFAD mice (*n* = 6 mice) and WT littermates (*n* = 6 mice). Differential expression *p*-values were calculated with nCounter Advanced Analysis and are adjusted for false discovery rate (Benjamini–Yekutieli method), ∗*p* < 0.05, ∗∗∗*p* < 0.001, ∗∗∗∗*p* < 0.0001. *E* and *F*, coimmunostaining for HSV-1 (gD antibody, *red*), reactive microglia (anti-Iba1 antibody, *green*), and nuclei (DAPI, *blue*) in motor cortices of 10-month-old 5XFAD mice infected IC with 10^4^ PFUs of McKrae and examined 24 h postinfection (*n* = 5 mice). *E*, and uninfected age-matched 5XFAD (*n* = 2 mice) (*F*).

The above results on lack of HSV-1 in Aβ aggregates led to consideration of an alternative hypothesis that reactive microglia and astrocytes in the brain areas with high density of Aβ aggregates are responsible for preventing spread of the virus to those areas. In normal brains, microglia are responsible for recognizing, engulfing, and phagocytosis of neurons infected with herpes viruses, a process that first requires activation of microglia (42). We proposed that in the areas with a high density of Aβ aggregates, microglia are already transitioned into a chronic reactive state and, as a result, are primed to phagocytose the virus. Indeed, coimmunostaining of 10-month-old uninfected 5XFADs using anti-Iba1 (marker of reactive microglia) and 6E10 antibodies confirmed the presence of reactive microglia in areas with high density of extracellular Aβ aggregates (Fig. 11*A*). In fact, in motor cortices, reactive microglia were found primarily in areas with high densities of Aβ aggregation, but considerably less frequent in areas that lacked extracellular Aβ deposition (Fig. 11*A*). Consistent with previous studies of the 5XFAD model (43, 44), individual Aβ aggregates were surrounded by several reactive microglia cells with processes spreading and penetrating into Aβ deposits, along with one or few reactive astrocytes (Fig. 11*B*). As expected, reactive microglia and astrocytes were lacking in uninfected age-matched WT littermates (Fig. 11*C*).

Next, for testing whether microglia are primed for phagocytosis, we analyzed expression of microglia- and astrocyte-specific genes, which report on neuroinflammation, in brain areas with high density Aβ aggregates, cortex and hippocampus, in 10-month-old noninfected animals (Fig. 11*D*). Both regions showed upregulation of genes associated with reactive microglia and astrocytes (Fig. 11*D*). Of particular interest was the upregulation of two microglia-specific genes *CD68*, a lysosomal receptor involved in phagocytosis (45), and *P2RY12*, a chemoreceptor for adenosine diphosphate. The receptor encoded by *P2RY12* senses a broad range of CNS insults including viral infections and drives microglia into a phagocytic state (Fig. 11*D*) (42, 46, 47). Upregulation of these markers supports the idea that in the brain areas with heavy deposition of Aβ aggregates reactive microglia are primed for phagocytic activity.

During the peak of the acute HSE (3–8 days postinfection), we routinely observed microglia engulfing neuronal cells that were actively replicating HSV-1 (Fig. 3*B*). For testing whether reactive microglia in aged 5XFAD phagocytose HSV-1 particles at the early stages of infection, *i.e.*, during its spread, 10-month-old 5XFADs were examined 24 h postinfection using coimmunostaining with anti-Iba1 and gD antibodies. In agreement with the idea that reactive microglia limit the spread of HSV-1, microglia that engulf HSV-1 particles were observed in areas of the motor cortex characterized by a high density of Aβ aggregates (Fig. 11*E*).

## Discussion

Contrary to previous studies (7), the 5XFAD genotype was not protective against acute HSV-1 infection in the current work. This conclusion is supported by experiments that employed two HSV-1 strains, 17syn+ and McKrae, each administered at three different doses into young 5XFAD mice, as well as by the examination of the survival rate of two aged 5XFAD cohorts upon challenge with McKrae. In support of this conclusion, expression of the APP variant associated with familial AD did not alter the region-specific or cell-specific tropisms of HSV-1 strains in 5XFAD mice. These results suggest that the major events in the host–pathogen interaction, such as HSV-1 trafficking, cell invasion or replication, are not affected by APP overexpression. In fact, active sites of HSV-1 replication were found in both APP-positive and -negative neurons. Also contrary to the previous studies (7), in the current work, young 5XFAD mice that survived infection cleared HSV-1 virus in the brain areas susceptible to virus replication without triggering Aβ aggregation. In aged mice, the brain areas with high density of Aβ aggregates were largely protected from HSV-1 spread and replication. However, no evidence of viral entrapment by preexisting extracellular Aβ aggregates was found in aged animals. Instead, the protection effect was attributed to reactive microglia, which were primarily localized in the areas with intensive extracellular Aβ deposition and were primed for phagocytic activity.

The discrepancy between the current and previous studies could be due to the substantially higher dose of HSV-1 used in the previous work (7) compared with the current study or to differences in the strain of HSV-1 used. In the current work, young mice were challenged with three doses of McKrae or 17syn+, which were several-fold above or below the strain-specific LD_50_ values. An LD_50_ value depends on both the HSV-1 strain and the mouse strain. Previous studies administered 2 × 10^7^ PFUs per mouse (7), a dose that was 10^2^- to 10^4^-fold higher than the doses in the current work. It is difficult to estimate how high above the LD_50_ value the 2 × 10^7^ PFUs was, because information regarding the HSV-1 strain was not provided. Thus, there is a possibility that the 5XFAD genotype is indeed protective against HSV-1, yet under a narrower set of experimental conditions, *i.e.*, a specific dose or strain of HSV-1. Another experimental parameter that differed from the previous study was the sex of animals. In the current work, both males and females were used in a random ratio in each experiment, whereas the previous study tested only females (7). Indeed, female 5XFAD mice generate higher levels of Aβ peptides relative to males (32, 34). However, analysis of the survival curves for females in the current work also showed a lack of differences between 5XFAD and WT cohorts.

In the current work, young 5XFAD mice that survived acute HSE cleared HSV-1 infection without triggering extracellular Aβ aggregation in the brain areas susceptible to HSV-1 invasion. During acute stages of HSE, reactive microglia were found in the sites of active HSV-1 replication and often seen engulfing infected neurons (Fig. 3*B*). These results are consistent with the well-established views on the role of microglia in clearance of viral infections (42, 48). In contrast to our results, in the previous work, HSV-1 was found to colocalize with Aβ plaques 3 weeks postinfection in young 5XFAD mice that survived the challenge (7). It is not clear whether apparent colocalization was due to actual preferences of HSV-1 for binding to Aβ or overloading of a brain with very high, nonphysiological doses of HSV-1. Possible cross-reactivity of anti-Aβ antibodies to HSV-1 along with a lack of negative controls could also account for apparent colocalization of HSV-1 with Aβ.

In the current work, 7- to 10-month-old 5XFAD animals, in which extracellular Aβ aggregates were abundant, showed a longer survival and a slightly better survival rate relative to the WT mice, although, the differences between the two groups were not statistically significant (Fig. 5*A*). The modest differences were abolished in older, 12- to 15-month-old animals, arguing that the weak protective effect was not attributed to the load of Aβ aggregates. Surprisingly, in 7- to 10-month-old 5XFAD mice, HSV-1 was found to be primarily excluded from brain areas with abundant extracellular Aβ aggregation (Fig. 5, *C* and *D*). This result prompted us to examine entrapment of HSV-1 by Aβ aggregates. Antibodies to three glycoproteins (gB, gD, and gH) of the HSV-1 envelope were employed for examining colocalization in 5XFAD mice that succumbed to acute HSE. Two antibodies (gD and gH) that were very effective in detecting the virus in infected neurons and brain parenchyma (Fig. S5) unambiguously showed a lack of colocalization between HSV-1 and Aβ aggregates. Moreover, no colocalization between HSV-1 and Aβ aggregates was observed during the early stages of infection (24–48 h postinoculation) or acute HSE (4–8 days postinoculation) (Figure 8, Figure 9, Figure 10). If the lack of colocalization was due to blocking of both gD and gH by Aβ, both epitopes should have remained 100% blocked, which is highly unlikely, especially considering that both gD and gH were effective in staining intracellular HSV-1. The results obtained with an anti-gB antibody (clone T111) were not consistent between individual animals within the infected 5XFAD cohort. Coimmunostaining of Aβ aggregates in aged 5XFAD mice lacking HSV-1 revealed cross-reactivity of the clone T111 with Aβ aggregates. We do not know whether the cross-reactivity of the clone T111 depends on the biological age of aggregates, an aggregate-specific Aβ conformation, or Aβ_42/40_ ratios within individual aggregates or animals. Our data indicate that the results with the clone T111 are clearly attributable to its cross-reactivity with Aβ. This cross-reactivity is not surprising, considering that the gB protein has a segment homologous to the Aβ peptide. In summary, with respect to binding of HSV-1 by Aβ aggregates, our work arrives at the opposite conclusion from that of Eimer *et al.* (7). Our examination of negative controls (uninfected aged 5XFAD mice) helped to arrive at this conclusion.

The results on the limited spread of the virus to brains areas with the heavy deposition of Aβ aggregates can be explained by phagocytic activity of reactive microglia. Indeed, consistent with previous studies (43, 44), in aged animals, reactive microglia were found primarily in the areas with high densities of Aβ aggregates and in close proximity, or in tight association, with Aβ plaques. As evident from gene expression analysis and consistent with previous studies (49), reactive microglia were primed for phagocytic activity (Fig. 11). Moreover, microglia engulfing HSV-1 were observed in motor cortices at the very early stages of infection (Fig. 11). Lack of the partial protection effects in the oldest, 12- to 15-month-old 5XFAD group (Fig. 5*B*) could be due to reduced phagocytic activity of senescent microglia or gradual death of plaque-associated microglia (50, 51, 52).

The current work supports the well-established views that microglia are responsible for clearing viral infections and illustrates that fighting of viral infections relies on two strategies. The first one involves direct phagocytosis of HSV-1. This strategy is effective when microglia are already primed or activated and takes place at the early stages of CNS infection. The second strategy involves phagocytosis of infected neurons. Indeed, we routinely observed microglia engulfing HSV-1-infected neurons during the acute stages of HSE, *i.e.*, 4 to 8 days postinoculation (Fig. 3*B*). Activation of phagocytic activity in resting or homeostatic microglia is a multistep process, in which sensing of infected neurons *via* signaling through the P2Y12 receptor is key (42).

While the current findings do not support the antiviral role of Aβ, this work does not refute the viral hypothesis behind the etiology of late-onset AD. In fact, our observed lack of a protective effect of the 5XFAD genotype against acute HSV-1 infection does not report on the potential role of Aβ in latent or chronic HSV-1 infections, which are likely more relevant to the etiology of late-onset AD. Viral or microbial pathogens of the CNS could increase the risk of late-onset AD *via* multiple, Aβ-dependent or independent mechanisms. Persistent activation of microglia as a result of recurrent reactivation of latent infection or repetitive exposure to viruses might lead to chronic neuroinflammation, which is an important driver of chronic neurodegeneration. It is plausible that recurrent reactivation of latent HSV-1 infections over a lifetime would eventually result in chronic activation of microglia leading to development of Aβ plaques along with substantial neuronal loss. Indeed, recent studies demonstrated that sustained depletion of microglia in the 5XFAD model impaired plaque development, revealing a central role of reactive microglia in genesis of Aβ plaques (43). Moreover, microglia were found to promote dense-core plaques *via* phagocytic uptake of loosely organized Aβ and condensing it into dense deposits in lysosomes (53). It would be interesting to test whether the reactive phenotypes acquired by glia as a result of multiple exposures to HSV-1, or by recurrent reactivation of latent infections, are similar to those observed in late-onset AD.

Using a comprehensive set of experimental conditions, the current work argues against the hypothesis that Aβ exhibits a general antiviral effect. Two cohorts of aged 5XFAD animals, in which Aβ aggregates were abundant, did not show better protection against HSV-1 relative to the control mice. Moreover, in aged 5XFAD mice, Aβ aggregates were free of HSV-1, suggesting that preformed aggregates do not entrap the virus. While the current work does not confirm the antiviral role of Aβ, this study does not support nor refutes the hypothesis on the viral etiology of late-onset AD. Alternative hypotheses that do not rely on direct antiviral effects of Aβ, but instead involve chronic activation of microglia, should be considered in order to provide experimental support for advancing the viral etiology hypothesis of AD and explain the clear epidemiological findings.

## Experimental procedures

### Reagents or resources

Reagents or resources are listed in Table S1.

### Experimental models

Male and female 5XFAD mice [B6SJL-Tg(APPSwFlLon, PSEN1∗M146L∗L286V) 6799Vas/Mmjax] and WT littermates were used for inoculation experiments. 5XFAD mice and WT littermates were group-housed together in random ratios, 3 to 5 mice per cage, and kept on a 12 h light/dark cycle. The ratio of males and females for both 5XFAD and WT littermates was random in each experiment. The study was carried out in accordance with the recommendations in the Guide for the Care and Use of Laboratory Animals of the National Institutes of Health. The animal protocol was approved by the Institutional Animal Care and Use Committee of the University of Maryland, Baltimore (Assurance Number: A32000-01; Permit Number: 0419007).

### Production and titration of HSV-1 stocks

HSV-1 17Syn+ and McKrae strains were propagated in Vero cell culture with initial multiplicity of infection (MOI) of 0.01 PFU/cell in serum-free medium. After 2 h of incubation at 37 °C, the viral inoculum was aspirated, and the cell culture was supplemented with fresh DMEM medium with 5% newborn calf serum. Cells were incubated for 2 to 3 days, until 100% of the cells displayed cytopathic effects. After one freezing and thawing cycle, cells were sonicated five times for 30 s using a Misonix S-4000 sonicator at 600 W output, letting the cell suspension cool on ice for 1 min between sonications. After centrifugation of the Vero cell lysate at 12,000*g* for 10 min at 4 °C, virus-containing supernatant was mixed with 10% sterile BSA solution to a final concentration of 2% BSA and supplemented with 10× PBS to a final concentration of 1× PBS (with 137 mM of NaCl and 3 mM KCl). Viral stock was split into smaller aliquots in screw-capped cryovials and stored at −80 °C. The HSV-1 17 Syn+ strain was a generous gift of Dr Krause (FDA), and the HSV-1 McKrae strain was a generous gift of Dr Cohen (NIH).

Viral titer was measured by a standard plaque assay on Vero cell culture under 0.9% agar layer. Tenfold serial dilutions in 1 ml serum-free medium were added to the corresponding well of a 6-well plate in duplicates and incubated for 2 h with swirling every 30 min. Plates underwent plaque count after 2 to 3 days of incubation. Cells were fixed with methanol and stained with 0.5% Crystal Violet in 25% methanol for plaque visualization. To determine the titer of the stock, the number of plaques in duplicate was averaged for a given dilution.

### Bioassay

5XFAD APP/PS1 transgenic mice (32) overexpress FAD mutant forms of human APP (the Swedish mutation: K670N, M671L; the Florida mutation: I716V; the London mutation: V717I) along with PS1 (M146L; L286V) transgenes under the transcriptional control of the neuron-specific mouse Thy1 promoter (Tg6799 line) and produce elevated levels of Aβ42 peptide. 5XFAD lines (with B6SJLF1/J genetic background) were purchased from Jackson Laboratory and maintained by crossing hemizygous 5XFAD transgenic males with B6SJLF1/J female breeders. All pups were genotyped using Transnetyx genotypic services. All 5XFAD transgenic mice were hemizygous with respect to the transgene.

The ratios of males to females for both 5XFAD and WT littermates in each experiment were random. 5XFAD and WT littermates were group-housed together in random ratios, 3 to 5 mice per cage for the entire experiment. Control animals were caged separately from the animals inoculated with HSV-1. 17Syn+ and McKrae inoculation stocks were prepared in PBS containing 137 mM of NaCl, 3 mM KCl, and 2% sterile BSA and supplemented with antibiotics-antimycotics concentrate (Invitrogen). Immediately before inoculation, stocks were diluted with PBS-NaCl-KCl-BSA sterile solution to the required titer. Each 5XFAD or WT littermate control mouse received a single 20 μl of inoculum intracerebrally under 2% isoflurane anesthesia, in the center of the left hemisphere 2 mm away from the sagittal suture. The injection depth of 2 mm was achieved using a needle length stopper made from a needle cap. All animals handled IC injections well and fully recovered within 1 h. After inoculation, animals were weighed and observed daily for the following signs of acute herpes simplex virus encephalitis: eye and face inflammation, walking difficulty, tremors, hunched posture, and weight loss. The animals were euthanized when they demonstrated the severe aforementioned clinical signs and/or 20% loss of their weight, as determined relative to weights measured prior to virus inoculation.

### Histopathology and immunofluorescence

After euthanasia by CO_2_ asphyxiation, brains were immediately extracted and kept ice-cold during dissection. Brains were sliced using a rodent brain slicer matrix (Zivic Instruments). 3 mm central coronal sections of each brain were formalin-fixed and embedded in paraffin. 4 μm sections were produced using a Leica RM2235 microtome (Leica Biosystems), mounted on slides, and processed for immunohistochemistry.

Rehydrated slides were subjected to the procedure of epitope exposure that involved hydrated autoclaving at 121 °C for 20 min in trisodium citrate buffer, pH 6.0, with 0.05% Tween 20. All antibodies used in this study are listed in the Key Resources Table. Immunofluorescence detection was performed with AlexaFluor-488, AlexaFluor-546, or DyLight-350 labeled secondary antibodies. An autofluorescence eliminator (Sigma-Aldrich) was used according to the original protocol to reduce background fluorescence. Fluorescent images were collected using an inverted Nikon Eclipse TE2000-U microscope (Nikon Instruments Inc) equipped with an illumination system X-cite 120 (EXFO Photonics Solutions Inc) and a cooled 12 bit CoolSnap HQ CCD camera (Photometrics). Images were processed using WCIF ImageJ software (National Institute of Health). In triple staining images, DyLight-350 signal was artificially colored as white for better visualization.

### Analysis of viral genome number

Frontal and rear brain parts remaining after a central 3 mm coronal cut were separated into right and left halves and kept frozen at −80 °C for subsequent DNA isolation. For analysis of viral copy number, the left frontal portions of brains were used. DNA isolation was performed according to the manufacturer’s instructions using QIAamp DNA Mini Kit (QIAGEN). Copy numbers of HSV-1 were measured using Virusys Corporation’s HSV-1 qPCR kit with a FAM/BHQ-labeled probe specific for glycoprotein D (gD) of HSV-1 (Virusys, cat. # H1K240). To build a calibration curve for determining an absolute copy number, serial 1/10 dilutions of the Virusys kit standard were made that produced linear dependence within a range of 10^2^ to 10^8^ copies per reaction. PCR product was detected by CFX96 Real-Time PCR Detection System (Bio-Rad).

### Quantification of Aβ aggregates and HSV-1 replication centers

Images were taken under 10× magnification and the same exposure time. For quantification of Aβ aggregates and HSV-1 replication centers, the images taken in green (anti-Aβ) and red (anti-HSV-1) channels were converted to 8 bit grayscale and then subjected to ImageJ signal area measurement after automated thresholding. On images taken in the red channel, nonspecific spots arising from blood vessels were deleted prior to analysis.

### Analysis of gene expression by nanostring

After euthanasia by CO_2_ asphyxiation, brains were immediately extracted and kept ice-cold during dissection. Brains were sliced using a rodent brain slicer matrix (Zivic Instruments). Cortex and hippocampus samples were collected from 2 mm central coronal sections of each brain. RNA isolation was performed as described before (54). RNA samples were processed by the Institute for Genome Science at the University of Maryland School of Medicine using the nCounter custom-designed Nanostring gene panel (Nanostring Technologies). Only samples with an RNA integrity number RIN >7.2 were used for nanostring analysis. All data passed quality control, with no imaging, binding, positive control, or CodeSet content normalization flags. The analysis of data was performed using nSolver Analysis Software 4.0 and nCounter Advanced Analysis 2.0.115. Ten house-keeping genes (*Xpnpep1*, *Lars*, *Tbp*, *Mto1*, *Csnk2a2*, *CCdc127*, *Fam104a*, *Aars*, *Tada2b*, *Cnot10*) were used for normalization of gene expression.

### Quantification and statistical analysis

Statistical analysis of Figures 1 and 5, *A* and *B* was performed using GraphPad Prism software, versions 8.4.2 for Windows (GraphPad Software). Survival curves were compared using Log-rank (Mantel-Cox) test. Differences with the *p* values >0.05 were considered lacking statistical significance. The differences in integrated density of Aβ and HSV-1 signals in WT and 5XFAD brains in Figure 5*E* were analyzed by an ordinary two-way ANOVA with Šidák’s multiple comparisons test.

Analysis of aggregates and virus colocalization was performed using the Colocalization Threshold plugin of ImageJ software (National Institute of Health). After determination of threshold by the Costes method (55), the thresholded Mander's Split Colocalization coefficients were calculated for each channel (red and green). The box-and-whisker plot of tM1 and tM2 values in Figure 7 was built using Excel. The midline denotes the median, x represents the mean, and the ends of the boxes denote the 25th and 75th percentiles. The whiskers extend from the ends of the box to the minimum value and maximum value. A data point was considered an outlier if it exceeded a distance of 1.5 times the IQR below the first quartile or 1.5 times the IQR above the third quartile.

## Data availability

All data are contained within the article and its supporting information.

## Supporting information

This article contains supporting information.

## Conflicts of interest

The authors declare that they have no conflict of interests with the contents of this article.

## Supporting information

Suppelmental Figures S1–S5 and Table S1

## References

[1] Lambert J.-C., Ibrahim-Verbaas C.A., Harold D., Naj A.C., Sims R., Bellenguez C., Jun G., DeStefano A.L., Bis J.C., Beecham G.W., Grenier-Boley B., Russo G., Thornton-Wells T.A., Jones N., Smith A.V. (2013). Meta-analysis of 74,046 individuals identifies 11 new susceptibility loci for Alzheimer's disease. Nat. Genet..

[2] Glenner G.G., Wong C.W. (1984). Alzheimer's disease: Initial report of the purification and characterization of a novel cerebrovascular amyloid protein. Biochem. Biophys. Res. Commun..

[3] Goate A., Chartier-Harlin M.C., Mullan M., Brown J., Crawford F., Fidani L., Giuffra L., Haynes A., Irving N., James L., Mant R., Newton P., Rooke K., Roques P., Talbot C. (1991). Segregation of a missense mutation in the amyloid precursor protein gene with familial Alzheimer's disease. Nature.

[4] Piacentini R., De Chiara G., Li Puma D.D., Ripoli C., Marcocci M.E., Garaci E., Palamara A.T., Grassi C. (2014). HSV-1 and Alzheimer’s disease: More than a hypothesis. Front. Pharmacol..

[5] Itzhaki R.F., Wozniak M.A., Appelt D.M., Balin B.J. (2004). Infiltration of the brain by pathogens causes Alzheimer’s disease. Neurobiol. Aging.

[6] De Chiara G., Marcocci M.E., Sgarbanti R., Civitelli L., Ripoli C., Piacentini R., Garaci E., Grassi C., Palamara A.T. (2012). Infectious agents and neurodegeneration. Mol. Neurobiol..

[7] Eimer W.A., Vijaya Kumar D.K., Navalpur Shanmugam N.K., Rodriguez A.S., Mitchell T., Washicosky K.J., György B., Breakefield X.O., Tanzi R.E., Moir R.D. (2018). Alzheimer’s disease-associated β-amyloid is rapidly seeded by herpesviridae to protect against brain infection. Neuron.

[8] Ezzat K., Pernemalm M., Pålsson S., Roberts T.C., Järver P., Dondalska A., Bestas B., Sobkowiak M.J., Levänen B., Sköld M., Thompson E.A., Saher O., Kari O.K., Lajunen T., Sverremark Ekström E. (2019). The viral protein corona directs viral pathogenesis and amyloid aggregation. Nat. Commun..

[9] Kumar D.K.V., Choi S.H., Washicosky K.J., Eimer W.A., Tucker S., Ghofrani J., Lefkowitz A., McColl G., Goldstein L.E., Tanzi R.E., Moir R.D. (2016). Amyloid-β peptide protects against microbial infection in mouse and worm models of Alzheimer’s disease. Sci. Transl. Med..

[10] Moir R.D., Lathe R., Tanzi R.E. (2018). The antimicrobial protection hypothesis of Alzheimer's disease. Alzheimers Dement..

[11] Cairns D.M., Rouleau N., Parker R.N., Walsh K.G., Gehrke L., Kaplan D.L. (2020). A 3D human brain–like tissue model of herpes-induced Alzheimer’s disease. Sci. Adv..

[12] Little C.S., Hammond C.J., MacIntyre A., Balin B.J., Appelt D.M. (2004). Chlamydia pneumoniae induces Alzheimer-like amyloid plaques in brains of BALB/c mice. Neurobiol. Aging.

[13] Hammond C.J., Hallock L.R., Howanski R.J., Appelt D.M., Little C.S., Balin B.J. (2010). Immunohistological detection of Chlamydia pneumoniae in the Alzheimer's disease brain. BMC Neurosci..

[14] Gérard H.C., Wildt K.L., Whittum-Hudson J.A., Lai Z., Ager J., Hudson A.P. (2005). The load of Chlamydia pneumoniae in the Alzheimer's brain varies with APOE genotype. Microb. Pathog..

[15] Little C.S., Joyce T.A., Hammond C.J., Matta H., Cahn D., Appelt D.M., Balin B.J. (2014). Detection of bacterial antigens and Alzheimer’s disease-like pathology in the central nervous system of BALB/c mice following intranasal infection with a laboratory isolate of Chlamydia pneumoniae. Front. Aging Neurosci..

[16] Miklossy J., Kis A., Radenovic A., Miller L., Forro L., Martins R., Reiss K., Darbinian N., Darekar P., Mihaly L., Khalili K. (2006). Beta-amyloid deposition and Alzheimer's type changes induced by Borrelia spirochetes. Neurobiol. Aging.

[17] Miklossy J. (2015). Historic evidence to support a causal relationship between spirochetal infections and Alzheimer’s disease. Front. Aging Neurosci..

[18] Tsai M.-C., Cheng W.-L., Sheu J.-J., Huang C.-C., Shia B.-C., Kao L.-T., Lin H.-C. (2017). Increased risk of dementia following herpes zoster ophthalmicus. PLoS One.

[19] Hogestyn J., Mock D., Mayer-Proschel M. (2018). Contributions of neurotropic human herpesviruses herpes simplex virus 1 and human herpesvirus 6 to neurodegenerative disease pathology. Neural Regen. Res..

[20] Harris S.A., Harris E.A. (2018). Molecular mechanisms for herpes simplex virus type 1 pathogenesis in Alzheimer’s disease. Front. Aging Neurosci..

[21] De Chiara G., Piacentini R., Fabiani M., Mastrodonato A., Marcocci M.E., Limongi D., Napoletani G., Protto V., Coluccio P., Celestino I., Li Puma D.D., Grassi C., Palamara A.T. (2019). Recurrent herpes simplex virus-1 infection induces hallmarks of neurodegeneration and cognitive deficits in mice. PLoS Pathog..

[22] Duarte L.F., Farías M.A., Álvarez D.M., Bueno S.M., Riedel C.A., González P.A. (2019). Herpes simplex virus type 1 infection of the central nervous system: Insights into proposed interrelationships with neurodegenerative disorders. Front. Cell Neurosci..

[23] Itzhaki R.F. (2018). Corroboration of a major role for herpes simplex virus type 1 in Alzheimer’s disease. Front. Aging Neurosci..

[24] Readhead B., Haure-Mirande J.-V., Funk C.C., Richards M.A., Shannon P., Haroutunian V., Sano M., Liang W.S., Beckmann N.D., Price N.D., Reiman E.M., Schadt E.E., Ehrlich M.E., Gandy S., Dudley J.T. (2018). Multiscale analysis of independent Alzheimer’s cohorts finds disruption of molecular, genetic, and clinical networks by human herpesvirus. Neuron.

[25] Jeong H.-H., Liu Z. (2019). Are HHV-6A and HHV-7 really more abundant in Alzheimer’s disease?. Neuron.

[26] Allnutt M.A., Johnson K., Bennett D.A., Connor S.M., Troncoso J.C., Pletnikova O., Albert M.S., Resnick S.M., Scholz S.W., De Jager P.L., Jacobson S. (2020). Human herpesvirus 6 detection in Alzheimer’s disease cases and controls across multiple cohorts. Neuron.

[27] Webre J.M., Hill J.M., Nolan N.M., Clement C., McFerrin H.E., Bhattacharjee P.S., Hsia V., Neumann D.M., Foster T.P., Lukiw W.J., Thompson H.W. (2012). Rabbit and mouse models of HSV-1 latency, reactivation, and recurrent eye diseases. J. Biomed. Biotechnol..

[28] Szpara M.L., Gatherer D., Ochoa A., Greenbaum B., Dolan A., Bowden R.J., Enquist L.W., Legendre M., Davison A.J. (2014). Evolution and diversity in human herpes simplex virus genomes. J. Virol..

[29] Sawtell N.M., Poon D.K., Tansky C.S., Thompson R.L. (1998). The latent herpes simplex virus type 1 genome copy number in individual neurons is virus strain specific and correlates with reactivation. J. Virol..

[30] Sawtell N.M., Thompson R.L. (1992). Rapid *in vivo* reactivation of herpes simplex virus in latently infected murine ganglionic neurons after transient hyperthermia. J. Virol..

[31] Kollias C.M., Huneke R.B., Wigdahl B., Jennings S.R. (2015). Animal models of herpes simplex virus immunity and pathogenesis. J. Neurovirol..

[32] Oakley H., Cole S.L., Logan S., Maus E., Shao P., Craft J., Guillozet-Bongaarts A., Ohno M., Disterhoft J., Van Eldik L., Berry R., Vassar R. (2006). Intraneuronal β-amyloid aggregates, neurodegeneration, and neuron loss in transgenic mice with five familial Alzheimer's disease mutations: Potential factors in amyloid plaque formation. J. Neurosci..

[33] Wang H., Davido D.J., Morrison L.A. (2014). HSV-1 strain McKrae is more neuroinvasive than HSV-1 KOS after corneal or vaginal inoculation in mice. Virus Res..

[34] Sadleir K.R., Eimer W.A., Cole S.L., Vassar R. (2015). Aβ reduction in BACE1 heterozygous null 5XFAD mice is associated with transgenic APP level. Mol. Neurodegener..

[35] Drayman N., Patel P., Vistain L., Tay S. (2019). HSV-1 single-cell analysis reveals the activation of anti-viral and developmental programs in distinct sub-populations. Elife.

[36] Braun E., Zimmerman T., Hur T.B., Reinhartz E., Fellig Y., Panet A., Steiner I. (2006). Neurotropism of herpes simplex virus type 1 in brain organ cultures. J. Gen. Virol..

[37] Reinert L.S., Lopušná K., Winther H., Sun C., Thomsen M.K., Nandakumar R., Mogensen T.H., Meyer M., Vægter C., Nyengaard J.R., Fitzgerald K.A., Paludan S.R. (2016). Sensing of HSV-1 by the cGAS–STING pathway in microglia orchestrates antiviral defence in the CNS. Nat. Commun..

[38] Cymerys J., Kowalczyk A., Mikotajewicz K., ajewicz K., Stonska A., Krzyzowska M. (2019). Nitric oxide influences HSV-1-induced neuroinflammation. Oxid. Med. Cell Longev..

[39] Villalba M., Hott M., Martin C., Aguila B., Valdivia S., Quezada C., Zambrano Á., Concha M.I., Otth C. (2012). Herpes simplex virus type 1 induces simultaneous activation of Toll-like receptors 2 and 4 and expression of the endogenous ligand serum amyloid A in astrocytes. Med. Microbiol. Immunol..

[40] Bello-Morales R., Praena B., de la Nuez C., Rejas M.T., Guerra M., Galán-Ganga M., Izquierdo M., Calvo V., Krummenacher C., López-Guerrero J.A. (2018). Role of microvesicles in the spread of herpes simplex virus 1 in oligodendrocytic cells. J. Virol..

[41] Dunn K.W., Kamocka M.M., McDonald J.H. (2011). A practical guide to evaluating colocalization in biological microscopy. Am. J. Physiol. Cell Physiol..

[42] Fekete R., Cserép C., Lénárt N., Tóth K., Orsolits B., Martinecz B., Méhes E., Szabó B., Németh V., Gönci B., Sperlágh B., Boldogkői Z., Kittel Á., Baranyi M., Ferenczi S. (2018). Microglia control the spread of neurotropic virus infection via P2Y12 signalling and recruit monocytes through P2Y12-independent mechanisms. Acta Neuropathol..

[43] Spangenberg E., Severson P.L., Hohsfield L.A., Crapser J., Zhang J., Burton E.A., Zhang Y., Spevak W., Lin J., Phan N.Y., Habets G., Rymar A., Tsang G., Walters J., Nespi M. (2019). Sustained microglial depletion with CSF1R inhibitor impairs parenchymal plaque development in an Alzheimer's disease model. Nat. Commun..

[44] Spangenberg E.E., Lee R.J., Najafi A.R., Rice R.A., Elmore M.R., Blurton-Jones M., West B.L., Green K.N. (2016). Eliminating microglia in Alzheimer's mice prevents neuronal loss without modulating amyloid-β pathology. Brain.

[45] Janda E., Boi L., Carta A.R. (2018). Microglial phagocytosis and its regulation: A therapeutic target in Parkinson’s disease?. Front. Mol. Neurosci..

[46] Haynes S.E., Hollopeter G., Yang G., Kurpius D., Dailey M.E., Gan W.-B., Julius D. (2006). The P2Y12 receptor regulates microglial activation by extracellular nucleotides. Nat. Neurosci..

[47] Hidetoshi T.-S., Makoto T., Inoue K. (2012). P2Y receptors in microglia and neuroinflammation. Wiley Interdiscip. Rev. Membr. Transp. Signal..

[48] Hatton C.F., Duncan C.J.A. (2019). Microglia are essential to protective antiviral immunity: Lessons from mouse models of viral encephalitis. Front. Immunol..

[49] Fricker M., Oliva-Martín M.J., Brown G.C. (2012). Primary phagocytosis of viable neurons by microglia activated with LPS or Aβ is dependent on calreticulin/LRP phagocytic signalling. J. Neuroinflammation.

[50] Yanguas-Casás N., Crespo-Castrillo A., Arevalo M.-A., Garcia-Segura L.M. (2020). Aging and sex: Impact on microglia phagocytosis. Aging Cell.

[51] Thériault P., Rivest S. (2016). Microglia: Senescence impairs clearance of myelin debris. Curr. Biol..

[52] Baik S.H., Kang S., Son S.M., Mook-Jung I. (2016). Microglia contributes to plaque growth by cell death due to uptake of amyloid β in the brain of Alzheimer's disease mouse model. Glia.

[53] Huang Y., Happonen K.E., Burrola P.G., O'Connor C., Hah N., Huang L., Nimmerjahn A., Lemke G. (2021). Microglia use TAM receptors to detect and engulf amyloid β plaques. Nat. Immunol..

[54] Makarava N., Chang J.C.-Y., Molesworth K., Baskakov I.V. (2020). Region-specific glial homeostatic signature in prion diseases is replaced by a uniform neuroinflammation signature, common for brain regions and prion strains with different cell tropism. Neurobiol. Dis..

[55] Costes S.V., Daelemans D., Cho E.H., Dobbin Z., Pavlakis G., Lockett S. (2004). Automatic and quantitative measurement of protein-protein colocalization in live cells. Biophys. J..

