# Supplementary material for: Alzheimer’s disease-associated β-amyloid does not protect against herpes simplex virus 1 infection in the mouse brain
Source: J Biol Chem. 2021 May 28;297(1):100845. doi: 10.1016/j.jbc.2021.100845 (PMC8214219; doi:10.1016/j.jbc.2021.100845)
Supplement: Suppelmental Figures S1–S5 and Table S1 [file mmc1.pdf]

Alzheimer's disease-associated β-Amyloid does not protect against Herpes Simplex Virus 1 infection in the mouse brain

Olga Bocharova[1,2], Narayan P. Pandit[1,2], Kara Molesworth[1,2], Aidan Fisher[1,2], Olga Mychko[1,2], Natallia Makarava[1,2], Ilia V. Baskakov[1,2*]

[1] Center for Biomedical Engineering and Technology, University of Maryland School of Medicine, Baltimore, MD, 21201, United States of America; [2]Department of Anatomy and Neurobiology, University of Maryland School of Medicine, Baltimore, MD, 21201, United States of America

Supporting Figure Legends

Figure S1. Dose-Response of Young Female 5XFAD Mouse Model to HSV-1 challenge. Survival curves for 5 to 6-weeks old female 5XFAD and wild-type littermate (WT) mice challenged via IC injections with 10^5, 10^4 or 10^3 PFUs of 17syn+ strain per mouse (**A**); or 10^4, $5x10^3$, or 10^3 PFUs of McKrae strain per mouse (**B**). 5XFAD and WT littermate mice were caged together in random ratios. Individual plots show independent experiments with number (*n*) of animals of each genotype indicated. Statistical significance (*p*) was calculated using the log-rank (Mantel-Cox) test.

Figure S2. Region-Specific Tropism of HSV-1 Is Not Altered in 5XFAD Mice. (A) Immunostaining for HSV-1 replication centers (a-HSV1 antibody, red) and cell nuclei (DAPI, blue) in 5XFAD (*n*=5 mice) and WT littermates (*n*=5 mice) that did not survive IC challenge with $5x10^3$ PFUs of McKrae. **(B)** Immunostaining for HSV-1 replication centers (a-HSV1 antibody, red) and cell nuclei (DAPI, blue) in 5XFAD mice that did not survive IC challenge with 10^4 PFUs of McKrae, or age-matched uninfected 5XFAD mice.

Figure S3. HSV-1 Does not Induce Formation of Aβ Aggregates in Young 5XFAD Mice. (A, B) Co-immunostaining for Aβ aggregates (6E10 antibody, green), HSV-1 replication centers (a-HSV1 antibody, red), and nuclei (DAPI, blue) in 5XFAD mice that survived IC challenge with 10^4 PFUs of 17syn+ (*n*=3 mice) (**A**) or 10^4 PFUs of McKrae (*n*=2 mice) (**B**). Animals were examined 2 weeks or 7.5 weeks upon IC challenge. No HSV-1 replication centers could be detected in animals that survived HSV-1 challenge. **(C)** Co-immunostaining for Aβ aggregates (6E10 antibody, green), HSV-1 replication centers (a-HSV1 antibody, red), and nuclei (DAPI, blue) in 4.5-month old (left panel, *n*=2 mice) and 11-month old (right panel *n*=2 mice) control 5XFAD mice. In control aged 5XFAD mice (**C**), 6E10 antibody stains both APP and Aβ aggregates (white arrows). However, in HSV-1-inoculated young 5XFAD mice (**A, B**), only intracellular APP staining can be seen.

Figure S4. Lack of Protective Effect of Aβ in Aged 5XFAD Mice. (**A**) Survival curves for 7 to10 month old female 5XFAD and WT littermate mice challenged IC with $5x10^3$ PFUs of McKrae per mouse. 5XFAD and WT mice were caged together in random ratios. Statistical significance (*p*) was calculated using the log-rank (Mantel-Cox) test. (**B**) Analysis of the number of HSV-1 genome copies in brains of WT (*n*=3 mice) and 5XFAD (*n*=4 mice) mice that where IC challenged with McKrae and reached terminal stage of acute encephalitis at 120 and 144 hours post-inoculation. Mean ± SD are shown; *n*=3 (WT); *n*=4 (5XFAD). The control group consists of age-matched non-inoculated WT and 5XFAD mice (combined *n*=4 mice).

Figure S5. Antibodies to Viral Replication Sites and Envelope Proteins gD and gH Stain the Same Cells and Brain Regions. Co-immunostaining for HSV-1 replication centers (a-HSV1 antibody, green) and viral envelope proteins gD or gH (anti-gD or anti-gH antibodies, red) in 6-week old 5XFAD (*n*=6 mice) infected IC with 10^4 PFUs of McKrae.

Figure S1

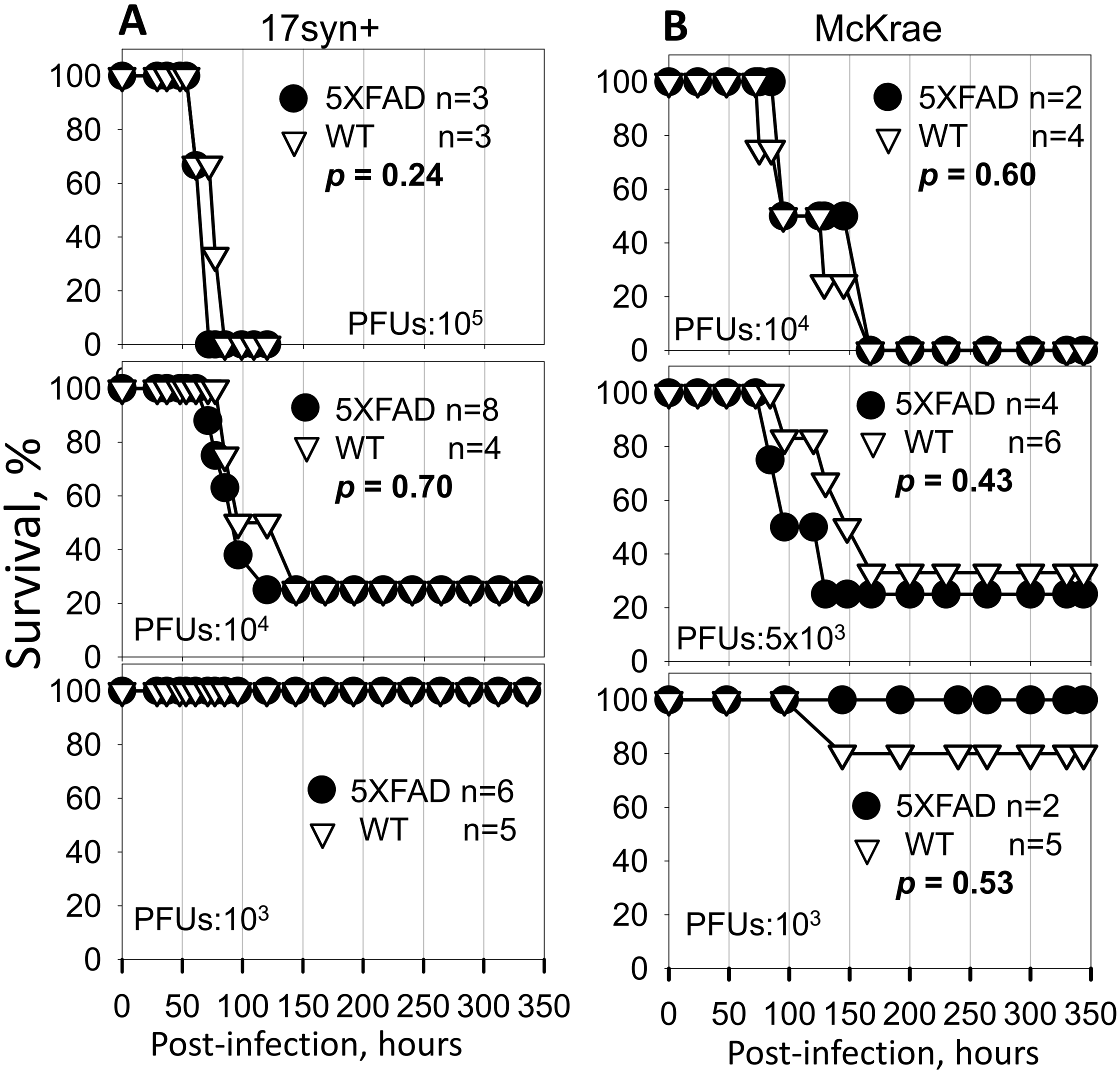
A
17syn+
B
McKrae
5XFAD n=3
WT n=3
p = 0.24
PFUs:10^5
5XFAD n=2
WT n=4
p = 0.60
PFUs:10^4
5XFAD n=8
WT n=4
p = 0.70
PFUs:10^4
5XFAD n=4
WT n=6
p = 0.43
PFUs:5x10^3
5XFAD n=6
WT n=5
PFUs:10^3
5XFAD n=2
WT n=5
p = 0.53
PFUs:10^3
Survival, %
0 50 100 150 200 250 300 350
Post-infection, hours
Post-infection, hours

Figure S2

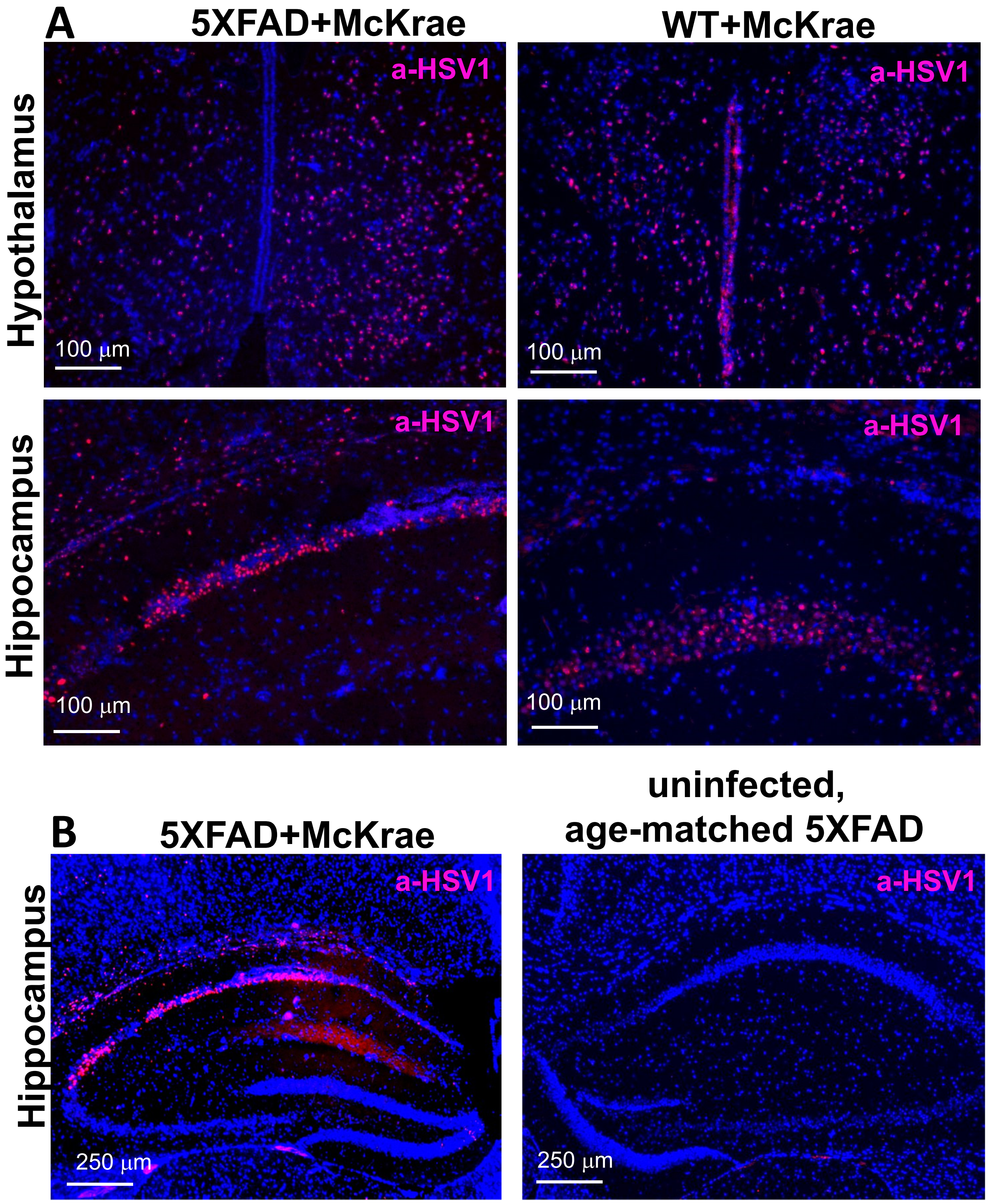
A
5XFAD+McKrae
WT+McKrae
Hypothalamus
Hippocampus
a-HSV1
100 µm
B
5XFAD+McKrae
uninfected,
age-matched 5XFAD
Hippocampus
a-HSV1
250 µm

Figure S3

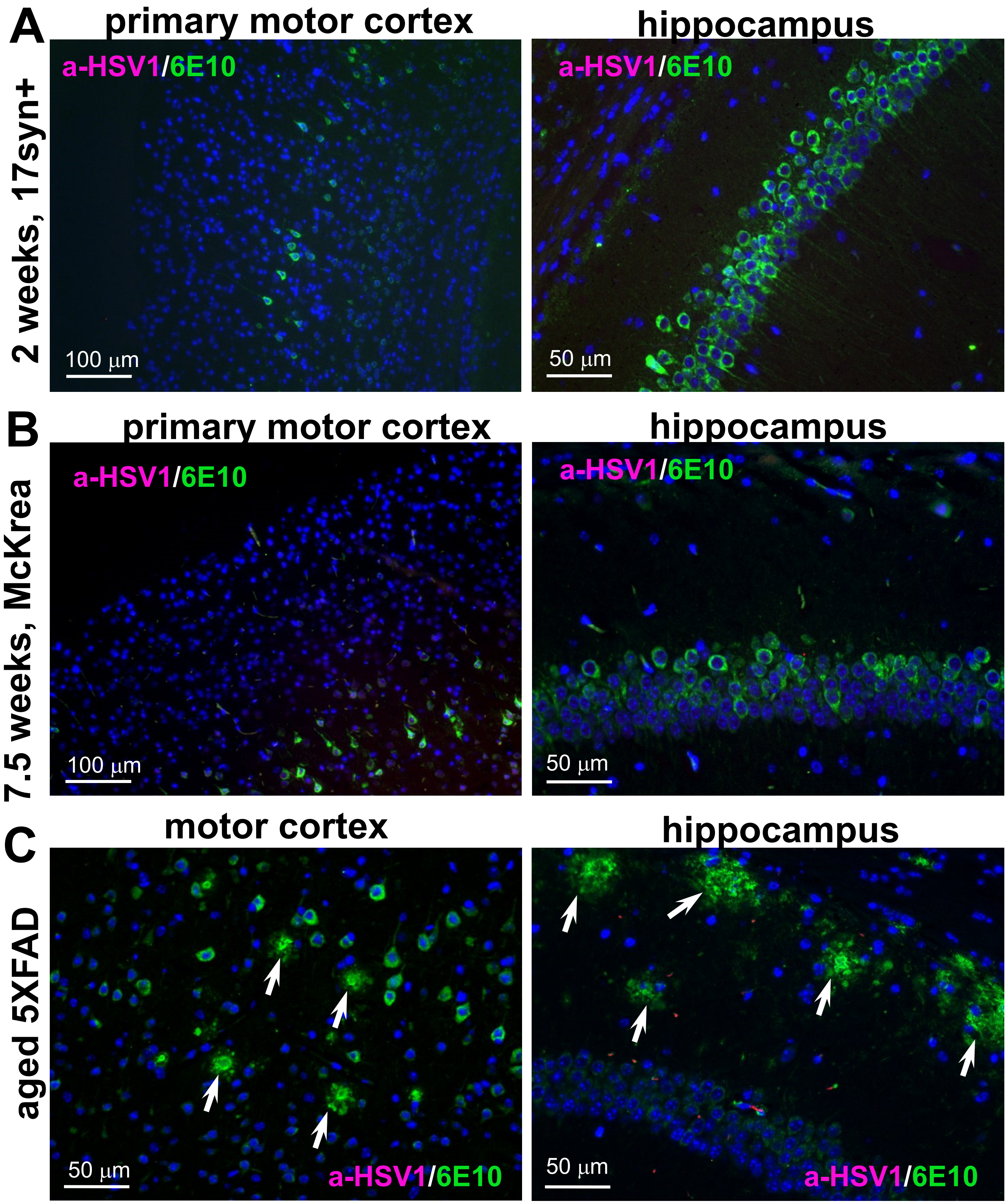

A
primary motor cortex
hippocampus
2 weeks, 17syn+
a-HSV1/6E10
a-HSV1/6E10
100 µm
50 µm
B
primary motor cortex
hippocampus
7.5 weeks, McKrea
a-HSV1/6E10
a-HSV1/6E10
100 µm
50 µm
C
motor cortex
hippocampus
aged 5XFAD
50 µm
a-HSV1/6E10
50 µm
a-HSV1/6E10

Figure S4

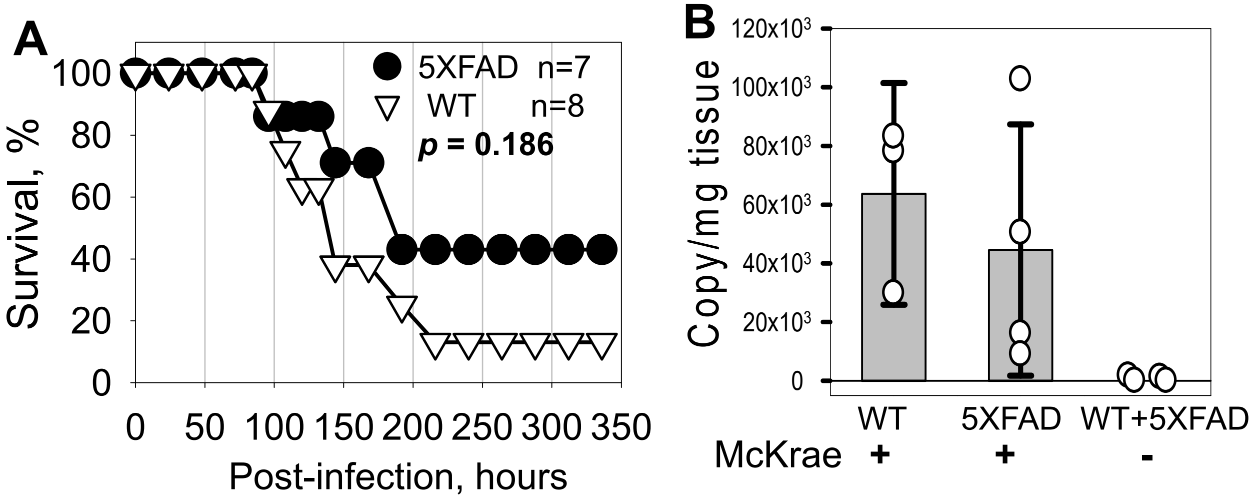

A
B
Survival, %
100
80
60
40
20
0
0
50
100
150
200
250
300
350
Post-infection, hours
5XFAD n=7
WT n=8
p = 0.186
Copy/mg tissue
120x10³
100x10³
80x10³
60x10³
40x10³
20x10³
0
WT
5XFAD
WT+5XFAD
McKrae
+
+
-

Figure S5

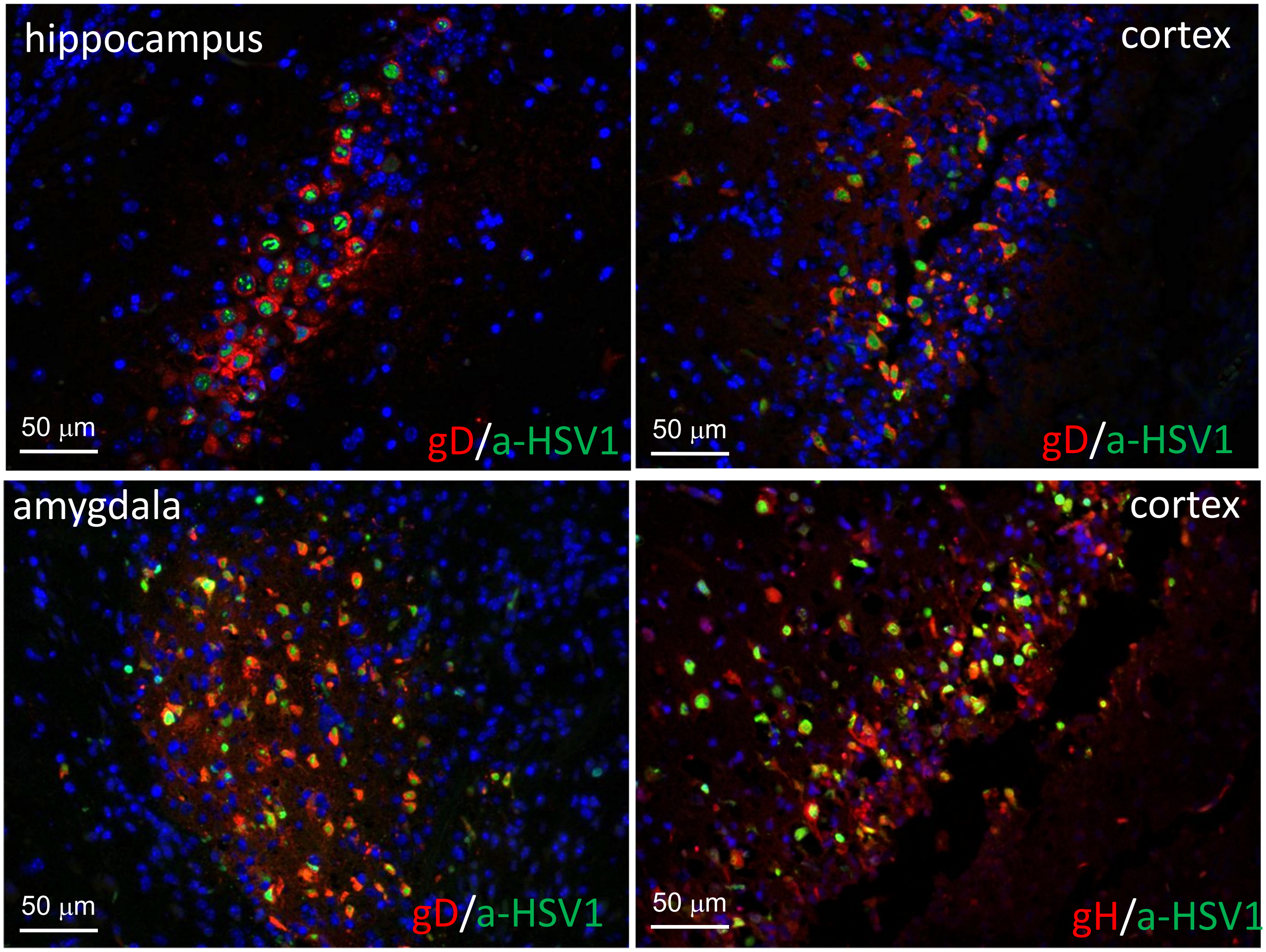
hippocampus
50 μm
gD/a-HSV1
cortex
50 μm
gD/a-HSV1
amygdala
50 μm
gD/a-HSV1
cortex
50 μm
gH/a-HSV1

Table S1. Reagents and Resources

Reagent or Resource	Source	Catalog #
**Antibodies**		
a-HSV Type 1/2 gB, clone T111	Sigma	SAB4700766
a-HSV1	Abcam	ab9533
a-β-Amyloid, 703-713, clone H31L21	Invitrogen	700254
a-HSV1/2 gD	Abcam	ab6507
a-HSV1 gH	Abcam	ab110227
a-β-Amyloid, 1-16, clone 6E10	Biolegend	803001
a-GFAP	Cell Signaling Technology	12389
a-GFAP	Millipore	AB5541
a-Iba1	FUJIFILM Wako Chemicals	019-19741
Goat a-mouse IgG (H+L) Alexa Fluor 546	Thermofisher Scientific	A11003
Goat a-rabbit IgG (H+L) Alexa Fluor 546	Thermofisher Scientific	A11010
Donkey a-mouse IgG (H+L) Alexa Fluor 488	Thermofisher Scientific	A21202
Donkey a-rabbit IgG (H+L) Alexa Fluor 488	Thermofisher Scientific	A21206
Goat a-chicken IgY (H+L)	Thermofisher Scientific	SA5-10069
**Virus strains**		
HSV-1 17 Syn+	Dr. Krause (FDA)	N/A
HSV-1 McKrae	Dr. Cohen (NIH)	N/A
**Cell lines**		
Vero	ATCC	RRID: CCL-81
**Experimental model**		
5XFAD mice	The Jackson Laboratory	34840-JAX
B6SJLF1/J mice	The Jackson Laboratory	100012
